# Supplementary material for: Universal Lineage-Independent Markers of Multidrug Resistance in Mycobacterium tuberculosis
Source: Microorganisms. 2024 Jun 30;12(7):1340. doi: 10.3390/microorganisms12071340 (PMC11278869; doi:10.3390/microorganisms12071340)
Supplement: Supplementary file 1 [file microorganisms-12-01340-s001.zip › Suppl. Table S1.pdf]

Suppl. Table S1. List of accession numbers of *M. tuberculosis* and Mtb-complex genomes at public depositaries: ERR###### – the European Nucleotide Archive (ENA); SRR####### – GenBank; TB#### – GMTV; and m#### or mal###### – BV-BRC

ERR067576	ERR067640	ERR067693	ERR067754	ERR108461	ERR117449	ERR133834	ERR133890
ERR067577	ERR067641	ERR067694	ERR067757	ERR108462	ERR117450	ERR133835	ERR133891
ERR067578	ERR067643	ERR067695	ERR067758	ERR108463	ERR117451	ERR133836	ERR133892
ERR067580	ERR067644	ERR067696	ERR067759	ERR108467	ERR117452	ERR133837	ERR133893
ERR067581	ERR067645	ERR067697	ERR067761	ERR108468	ERR117453	ERR133838	ERR133895
ERR067582	ERR067647	ERR067700	ERR067762	ERR108469	ERR117454	ERR133839	ERR133896
ERR067583	ERR067649	ERR067703	ERR067763	ERR108470	ERR117455	ERR133840	ERR133897
ERR067584	ERR067650	ERR067704	ERR067765	ERR108472	ERR117457	ERR133841	ERR133898
ERR067585	ERR067651	ERR067705	ERR067766	ERR108473	ERR117458	ERR133844	ERR133899
ERR067586	ERR067653	ERR067706	ERR067767	ERR108474	ERR117460	ERR133846	ERR133900
ERR067587	ERR067654	ERR067707	ERR108420	ERR108475	ERR117462	ERR133847	ERR133901
ERR067588	ERR067655	ERR067708	ERR108421	ERR108478	ERR117463	ERR133849	ERR133902
ERR067589	ERR067656	ERR067709	ERR108422	ERR108479	ERR117464	ERR133850	ERR133903
ERR067590	ERR067657	ERR067711	ERR108423	ERR108480	ERR117465	ERR133851	ERR133904
ERR067591	ERR067658	ERR067713	ERR108425	ERR108481	ERR117466	ERR133852	ERR133905
ERR067592	ERR067659	ERR067714	ERR108426	ERR108482	ERR117467	ERR133854	ERR133906
ERR067595	ERR067660	ERR067715	ERR108427	ERR108483	ERR117468	ERR133855	ERR133907
ERR067596	ERR067661	ERR067716	ERR108428	ERR108485	ERR117469	ERR133856	ERR133908
ERR067599	ERR067663	ERR067717	ERR108429	ERR108486	ERR117470	ERR133857	ERR133909
ERR067600	ERR067664	ERR067718	ERR108432	ERR108487	ERR133799	ERR133858	ERR133911
ERR067601	ERR067665	ERR067719	ERR108433	ERR108488	ERR133800	ERR133859	ERR133912
ERR067602	ERR067666	ERR067721	ERR108434	ERR108489	ERR133802	ERR133860	ERR133913
ERR067603	ERR067667	ERR067722	ERR108435	ERR108490	ERR133803	ERR133862	ERR133914
ERR067604	ERR067668	ERR067723	ERR108436	ERR108492	ERR133804	ERR133863	ERR133915
ERR067606	ERR067670	ERR067724	ERR108437	ERR108493	ERR133805	ERR133864	ERR133917
ERR067608	ERR067671	ERR067725	ERR108439	ERR108494	ERR133806	ERR133865	ERR133919
ERR067609	ERR067672	ERR067726	ERR108440	ERR108495	ERR133807	ERR133866	ERR133920
ERR067610	ERR067673	ERR067727	ERR108441	ERR108496	ERR133808	ERR133868	ERR133922
ERR067612	ERR067674	ERR067728	ERR108442	ERR108497	ERR133809	ERR133870	ERR133924
ERR067613	ERR067675	ERR067729	ERR108443	ERR108498	ERR133810	ERR133871	ERR133925
ERR067614	ERR067676	ERR067730	ERR108444	ERR108499	ERR133811	ERR133872	ERR133926
ERR067615	ERR067677	ERR067731	ERR108445	ERR108500	ERR133812	ERR133873	ERR133929
ERR067616	ERR067678	ERR067732	ERR108446	ERR108501	ERR133814	ERR133874	ERR133930
ERR067617	ERR067679	ERR067733	ERR108447	ERR108502	ERR133815	ERR133875	ERR133932
ERR067618	ERR067680	ERR067734	ERR108448	ERR108503	ERR133818	ERR133876	ERR133934
ERR067619	ERR067682	ERR067736	ERR108449	ERR108504	ERR133819	ERR133877	ERR133935
ERR067620	ERR067683	ERR067737	ERR108450	ERR108505	ERR133820	ERR133878	ERR133937
ERR067622	ERR067684	ERR067740	ERR108451	ERR108506	ERR133822	ERR133879	ERR133939
ERR067624	ERR067685	ERR067742	ERR108452	ERR108507	ERR133823	ERR133880	ERR133941
ERR067625	ERR067686	ERR067743	ERR108453	ERR108508	ERR133826	ERR133881	ERR133942
ERR067627	ERR067687	ERR067744	ERR108455	ERR108509	ERR133827	ERR133882	ERR133944
ERR067630	ERR067688	ERR067745	ERR108456	ERR108510	ERR133829	ERR133883	ERR133945
ERR067631	ERR067689	ERR067748	ERR108457	ERR108511	ERR133830	ERR133885	ERR133946
ERR067635	ERR067690	ERR067750	ERR108458	ERR108513	ERR133831	ERR133886	ERR133947
ERR067636	ERR067691	ERR067751	ERR108459	ERR108514	ERR133832	ERR133888	ERR133948
ERR067637	ERR067692	ERR067753	ERR108460	ERR108515	ERR133833	ERR133889	ERR133949

ERR133950	ERR137214	ERR137277	ERR144595	ERR158589	ERR228025	ERR229931	ERR229988
ERR133951	ERR137215	ERR137278	ERR144596	ERR158590	ERR228026	ERR229932	ERR229989
ERR133952	ERR137216	ERR137279	ERR144597	ERR158591	ERR228027	ERR229934	ERR229990
ERR133953	ERR137218	ERR137280	ERR144598	ERR158592	ERR228028	ERR229935	ERR229991
ERR133954	ERR137220	ERR137281	ERR144599	ERR158595	ERR228029	ERR229936	ERR229993
ERR133955	ERR137221	ERR137282	ERR144600	ERR158596	ERR228031	ERR229937	ERR229994
ERR133958	ERR137222	ERR137283	ERR144602	ERR158598	ERR228032	ERR229938	ERR229995
ERR133959	ERR137223	ERR137284	ERR144603	ERR158611	ERR228033	ERR229939	ERR229997
ERR133961	ERR137224	ERR137285	ERR144604	ERR158613	ERR228034	ERR229940	ERR229998
ERR133963	ERR137225	ERR144542	ERR144605	ERR158616	ERR228035	ERR229941	ERR229999
ERR133964	ERR137227	ERR144543	ERR144607	ERR227975	ERR228036	ERR229942	ERR230001
ERR133965	ERR137228	ERR144544	ERR144608	ERR227976	ERR228037	ERR229943	ERR230002
ERR133966	ERR137229	ERR144545	ERR144609	ERR227977	ERR228039	ERR229944	ERR230003
ERR133967	ERR137230	ERR144546	ERR144610	ERR227979	ERR228040	ERR229945	ERR230004
ERR133968	ERR137231	ERR144547	ERR144611	ERR227980	ERR228042	ERR229946	ERR230005
ERR133969	ERR137232	ERR144548	ERR144613	ERR227981	ERR228043	ERR229947	ERR230006
ERR133970	ERR137233	ERR144549	ERR144614	ERR227982	ERR228044	ERR229948	ERR230007
ERR133971	ERR137234	ERR144551	ERR144616	ERR227983	ERR228045	ERR229949	ERR230008
ERR133973	ERR137235	ERR144552	ERR144618	ERR227984	ERR228046	ERR229950	ERR230010
ERR133974	ERR137238	ERR144555	ERR144619	ERR227985	ERR228047	ERR229953	ERR234556
ERR133975	ERR137239	ERR144556	ERR144620	ERR227987	ERR228048	ERR229954	ERR234557
ERR133977	ERR137240	ERR144557	ERR144621	ERR227988	ERR228049	ERR229955	ERR234558
ERR133978	ERR137241	ERR144558	ERR144622	ERR227990	ERR228053	ERR229956	ERR234559
ERR133979	ERR137242	ERR144559	ERR144623	ERR227991	ERR228055	ERR229957	ERR234560
ERR133980	ERR137243	ERR144560	ERR144624	ERR227992	ERR228056	ERR229958	ERR234561
ERR133981	ERR137245	ERR144561	ERR144625	ERR227996	ERR228057	ERR229959	ERR234562
ERR133982	ERR137246	ERR144562	ERR144626	ERR227997	ERR228058	ERR229960	ERR234563
ERR133983	ERR137247	ERR144563	ERR144627	ERR227998	ERR228059	ERR229961	ERR234564
ERR133984	ERR137248	ERR144564	ERR144628	ERR227999	ERR228060	ERR229962	ERR234565
ERR133985	ERR137249	ERR144565	ERR144629	ERR228000	ERR228061	ERR229964	ERR234569
ERR133987	ERR137251	ERR144566	ERR144630	ERR228001	ERR228062	ERR229965	ERR234570
ERR133988	ERR137252	ERR144567	ERR144631	ERR228002	ERR228063	ERR229966	ERR234573
ERR133989	ERR137253	ERR144569	ERR144632	ERR228003	ERR228064	ERR229967	ERR234574
ERR137191	ERR137254	ERR144570	ERR144633	ERR228004	ERR228065	ERR229968	ERR234575
ERR137192	ERR137255	ERR144571	ERR144634	ERR228005	ERR228067	ERR229969	ERR234576
ERR137193	ERR137256	ERR144573	ERR144635	ERR228006	ERR228068	ERR229970	ERR234577
ERR137195	ERR137257	ERR144574	ERR144636	ERR228007	ERR228069	ERR229971	ERR234578
ERR137197	ERR137258	ERR144575	ERR158569	ERR228008	ERR229915	ERR229972	ERR234580
ERR137198	ERR137260	ERR144576	ERR158570	ERR228009	ERR229917	ERR229973	ERR234581
ERR137199	ERR137261	ERR144577	ERR158571	ERR228010	ERR229918	ERR229975	ERR234582
ERR137200	ERR137262	ERR144578	ERR158572	ERR228011	ERR229919	ERR229976	ERR234584
ERR137201	ERR137264	ERR144579	ERR158575	ERR228012	ERR229920	ERR229977	ERR234585
ERR137204	ERR137266	ERR144580	ERR158576	ERR228013	ERR229921	ERR229978	ERR234586
ERR137205	ERR137267	ERR144581	ERR158577	ERR228014	ERR229922	ERR229979	ERR234589
ERR137206	ERR137268	ERR144582	ERR158579	ERR228015	ERR229923	ERR229981	ERR234590
ERR137207	ERR137269	ERR144585	ERR158580	ERR228017	ERR229924	ERR229982	ERR234593
ERR137208	ERR137270	ERR144588	ERR158581	ERR228018	ERR229925	ERR229983	ERR234594
ERR137209	ERR137271	ERR144590	ERR158582	ERR228019	ERR229926	ERR229984	ERR234596
ERR137210	ERR137273	ERR144592	ERR158585	ERR228021	ERR229927	ERR229985	ERR234597
ERR137212	ERR137274	ERR144593	ERR158587	ERR228023	ERR229928	ERR229986	ERR234598
ERR137213	ERR137275	ERR144594	ERR158588	ERR228024	ERR229929	ERR229987	ERR234599

ERR234600	ERR234685	ERR550998	ERR551882	ERR552495	ERR553134	ERR2041716	ERR2041771
ERR234602	ERR234686	ERR551014	ERR551896	ERR552496	ERR553157	ERR2041717	ERR2041772
ERR234603	ERR234687	ERR551045	ERR551899	ERR552518	ERR553182	ERR2041718	ERR2041773
ERR234607	ERR234688	ERR551070	ERR551915	ERR552540	ERR553186	ERR2041719	ERR2041774
ERR234608	ERR234689	ERR551071	ERR551920	ERR552573	ERR553204	ERR2041720	ERR2041775
ERR234609	ERR234690	ERR551079	ERR551928	ERR552599	ERR553215	ERR2041721	ERR2041776
ERR234610	ERR234691	ERR551086	ERR551938	ERR552605	ERR553216	ERR2041722	ERR2041777
ERR234616	ERR234692	ERR551089	ERR551942	ERR552612	ERR553221	ERR2041723	ERR2041780
ERR234617	ERR234693	ERR551118	ERR551953	ERR552622	ERR553230	ERR2041724	ERR2041781
ERR234618	ERR234694	ERR551166	ERR551969	ERR552637	ERR553241	ERR2041727	ERR2041782
ERR234619	ERR234695	ERR551195	ERR551978	ERR552667	ERR553242	ERR2041728	ERR2041783
ERR234620	ERR234696	ERR551204	ERR551983	ERR552672	ERR553264	ERR2041729	ERR2041784
ERR234622	ERR234697	ERR551231	ERR552000	ERR552690	ERR553277	ERR2041730	ERR2041785
ERR234623	ERR234698	ERR551241	ERR552010	ERR552695	ERR553278	ERR2041731	ERR2041786
ERR234624	ERR234699	ERR551242	ERR552070	ERR552701	ERR553282	ERR2041732	ERR2041787
ERR234627	ERR498238	ERR551246	ERR552071	ERR552710	ERR553284	ERR2041733	ERR2041788
ERR234630	ERR498286	ERR551280	ERR552073	ERR552720	ERR553286	ERR2041734	ERR2041789
ERR234631	ERR498289	ERR551288	ERR552075	ERR552743	ERR553291	ERR2041735	ERR2041791
ERR234633	ERR498295	ERR551289	ERR552077	ERR552745	ERR553293	ERR2041736	ERR2041793
ERR234634	ERR498299	ERR551299	ERR552083	ERR552751	ERR553313	ERR2041738	ERR2041794
ERR234637	ERR498352	ERR551316	ERR552109	ERR552754	ERR553321	ERR2041739	ERR2041795
ERR234638	ERR498355	ERR551319	ERR552115	ERR552755	ERR553324	ERR2041741	ERR2041796
ERR234639	ERR498356	ERR551329	ERR552118	ERR552763	ERR553328	ERR2041742	ERR2041798
ERR234640	ERR498359	ERR551336	ERR552124	ERR552765	ERR553343	ERR2041743	ERR2041800
ERR234641	ERR498362	ERR551391	ERR552150	ERR552780	ERR553350	ERR2041744	ERR2041802
ERR234642	ERR498365	ERR551393	ERR552151	ERR552784	ERR553354	ERR2041745	ERR2041803
ERR234645	ERR498366	ERR551419	ERR552182	ERR552789	ERR553357	ERR2041746	ERR2041804
ERR234646	ERR498369	ERR551438-	ERR552193	ERR552806	ERR553358	ERR2041747	ERR2041805
ERR234647	ERR498384	ERR551440	ERR552221	ERR552809	ERR553366	ERR2041748	ERR2199742
ERR234648	ERR550619	ERR551456	ERR552246-	ERR552822	ERR553385	ERR2041749	ERR2199743
ERR234649	ERR550624	ERR551488	ERR552247	ERR552823	ERR619080	ERR2041750	ERR2199744
ERR234651	ERR550636	ERR551523	ERR552252	ERR552870	ERR2041683	ERR2041751	ERR2199745
ERR234652	ERR550657	ERR551536	ERR552254	ERR552878	ERR2041684	ERR2041752	ERR2199746
ERR234653	ERR550701	ERR551537	ERR552261	ERR552917	ERR2041685	ERR2041753	ERR2199749
ERR234654	ERR550712	ERR551545	ERR552267	ERR552929	ERR2041687	ERR2041754	ERR2199753
ERR234655	ERR550718	ERR551566	ERR552273	ERR552930	ERR2041688	ERR2041755	ERR2199754
ERR234657	ERR550734	ERR551572	ERR552288	ERR552981	ERR2041689	ERR2041756	ERR2199756
ERR234658	ERR550739	ERR551578	ERR552296	ERR552987	ERR2041691	ERR2041757	ERR2199761
ERR234660	ERR550741	ERR551609	ERR552314	ERR552992	ERR2041693	ERR2041758	ERR2199763
ERR234661	ERR550754	ERR551620	ERR552352	ERR552994	ERR2041695	ERR2041759	ERR2199765
ERR234662	ERR550794	ERR551621	ERR552361	ERR553000	ERR2041696	ERR2041760	ERR2199769
ERR234663	ERR550813	ERR551650	ERR552397	ERR553002	ERR2041698	ERR2041761	ERR2199770
ERR234666	ERR550861	ERR551675	ERR552405	ERR553021	ERR2041700	ERR2041762	ERR2199771
ERR234667	ERR550864	ERR551689	ERR552415	ERR553028	ERR2041702	ERR2041763	ERR2199772
ERR234668	ERR550868	ERR551692	ERR552420	ERR553053	ERR2041704	ERR2041764	ERR2199773
ERR234669	ERR550869	ERR551694	ERR552427	ERR553058	ERR2041706	ERR2041765	ERR2199775
ERR234671	ERR550872	ERR551739	ERR552456	ERR553059	ERR2041708	ERR2041766	ERR2199776
ERR234672	ERR550927	ERR551758	ERR552463	ERR553082	ERR2041709	ERR2041767	ERR2199777
ERR234674	ERR550942	ERR551801	ERR552484	ERR553105	ERR2041711	ERR2041768	ERR2199779
ERR234683	ERR550959	ERR551855	ERR552487	ERR553116	ERR2041712	ERR2041769	ERR2199780
ERR234684	ERR550976	ERR551857	ERR552491	ERR553125	ERR2041714	ERR2041770	ERR2199781

ERR2199782	ERR2199848	ERR2199913	ERR2199966	ERR2200024	ERR2200090	ERR2509673	ERR2510147
ERR2199783	ERR2199849	ERR2199914	ERR2199967	ERR2200025	ERR2200091	ERR2509674	ERR2510148
ERR2199784	ERR2199850	ERR2199915	ERR2199969	ERR2200027	ERR2200092	ERR2509675	ERR2510149
ERR2199786	ERR2199851	ERR2199916	ERR2199970	ERR2200028	ERR2200093	ERR2509676	ERR2510150
ERR2199787	ERR2199852	ERR2199917	ERR2199971	ERR2200029	ERR2200096	ERR2509677	ERR2510151
ERR2199788	ERR2199854	ERR2199918	ERR2199972	ERR2200032	ERR2200097	ERR2509678	ERR2510152
ERR2199789	ERR2199856	ERR2199919	ERR2199973	ERR2200033	ERR2200099	ERR2509679	ERR2510153
ERR2199791	ERR2199857	ERR2199920	ERR2199974	ERR2200034	ERR2200100	ERR2509680	ERR2510154
ERR2199792	ERR2199858	ERR2199921	ERR2199975	ERR2200035	ERR2200101	ERR2509681	ERR2510155
ERR2199794	ERR2199859	ERR2199922	ERR2199976	ERR2200036	ERR2200102	ERR2509682	ERR2510156
ERR2199795	ERR2199860	ERR2199923	ERR2199977	ERR2200037	ERR2200103	ERR2509683	ERR2510157
ERR2199796	ERR2199861	ERR2199924	ERR2199978	ERR2200038	ERR2200104	ERR2509684	ERR2510158
ERR2199798	ERR2199863	ERR2199925	ERR2199979	ERR2200039	ERR2200105	ERR2509685	ERR2510159
ERR2199799	ERR2199864	ERR2199926	ERR2199980	ERR2200040	ERR2200106	ERR2509686	ERR2510160
ERR2199800	ERR2199865	ERR2199927	ERR2199981	ERR2200041	ERR2200107	ERR2509879	ERR2510161
ERR2199801	ERR2199867	ERR2199928	ERR2199982	ERR2200042	ERR2200108	ERR2509880	ERR2510162
ERR2199803	ERR2199868	ERR2199929	ERR2199983	ERR2200043	ERR2200109	ERR2509881	ERR2510163
ERR2199804	ERR2199870	ERR2199930	ERR2199985	ERR2200044	ERR2200110	ERR2509882	ERR2510164
ERR2199806	ERR2199871	ERR2199931	ERR2199986	ERR2200045	ERR2200111	ERR2509883	ERR2510165
ERR2199807	ERR2199873	ERR2199932	ERR2199987	ERR2200049	ERR2200112	ERR2509884	ERR2510166
ERR2199808	ERR2199874	ERR2199933	ERR2199989	ERR2200050	ERR2200113	ERR2509885	ERR2510167
ERR2199809	ERR2199875	ERR2199934	ERR2199990	ERR2200051	ERR2200115	ERR2509886	ERR2510168
ERR2199810	ERR2199877	ERR2199935	ERR2199991	ERR2200052	ERR2200116	ERR2509887	ERR2510169
ERR2199812	ERR2199878	ERR2199936	ERR2199992	ERR2200053	ERR2200117	ERR2509888	ERR2510170
ERR2199815	ERR2199879	ERR2199937	ERR2199993	ERR2200054	ERR2200118	ERR2509889	ERR2510171
ERR2199816	ERR2199881	ERR2199938	ERR2199995	ERR2200055	ERR2200119	ERR2509890	ERR2510172
ERR2199817	ERR2199883	ERR2199939	ERR2199996	ERR2200056	ERR2200120	ERR2509891	ERR2510173
ERR2199818	ERR2199884	ERR2199940	ERR2199998	ERR2200057	ERR2200121	ERR2509892	ERR2510174
ERR2199819	ERR2199886	ERR2199941	ERR2199999	ERR2200059	ERR2200123	ERR2509893	ERR2510175
ERR2199820	ERR2199887	ERR2199942	ERR2200000	ERR2200062	ERR2200124	ERR2509894	ERR2510176
ERR2199821	ERR2199888	ERR2199943	ERR2200001	ERR2200063	ERR2200125	ERR2509895	ERR2510177
ERR2199822	ERR2199889	ERR2199944	ERR2200002	ERR2200065	ERR2200126	ERR2509896	ERR2510178
ERR2199823	ERR2199890	ERR2199945	ERR2200003	ERR2200067	ERR2200127	ERR2509897	ERR2510179
ERR2199825	ERR2199891	ERR2199946	ERR2200004	ERR2200069	ERR2200129	ERR2510129	ERR2510180
ERR2199827	ERR2199892	ERR2199947	ERR2200005	ERR2200070	ERR2200130	ERR2510130	ERR2510181
ERR2199828	ERR2199893	ERR2199948	ERR2200006	ERR2200071	ERR2200131	ERR2510131	ERR2510182
ERR2199830	ERR2199894	ERR2199949	ERR2200007	ERR2200073	ERR2200132	ERR2510132	ERR2510184
ERR2199831	ERR2199895	ERR2199950	ERR2200008	ERR2200074	ERR2200133	ERR2510133	ERR2510185
ERR2199832	ERR2199896	ERR2199951	ERR2200009	ERR2200076	ERR2200134	ERR2510134	ERR2510186
ERR2199833	ERR2199897	ERR2199952	ERR2200010	ERR2200077	ERR2200136	ERR2510135	ERR2510187
ERR2199834	ERR2199898	ERR2199954	ERR2200012	ERR2200078	ERR2200138	ERR2510136	ERR2510188
ERR2199835	ERR2199900	ERR2199955	ERR2200013	ERR2200079	ERR2200139	ERR2510137	ERR2510189
ERR2199837	ERR2199902	ERR2199956	ERR2200014	ERR2200080	ERR2200140	ERR2510138	ERR2510190
ERR2199838	ERR2199903	ERR2199957	ERR2200015	ERR2200081	ERR2200141	ERR2510139	ERR2510191
ERR2199839	ERR2199904	ERR2199958	ERR2200016	ERR2200082	ERR2200143	ERR2510140	ERR2510192
ERR2199840	ERR2199905	ERR2199960	ERR2200017	ERR2200084	ERR2200144	ERR2510141	ERR2510193
ERR2199841	ERR2199906	ERR2199961	ERR2200018	ERR2200085	ERR2200145	ERR2510142	ERR2510194
ERR2199842	ERR2199908	ERR2199962	ERR2200019	ERR2200086	ERR2200147	ERR2510143	ERR2510195
ERR2199845	ERR2199909	ERR2199963	ERR2200020	ERR2200087	ERR2200148	ERR2510144	ERR2510196
ERR2199846	ERR2199911	ERR2199964	ERR2200021	ERR2200088	ERR2200149	ERR2510145	ERR2510197
ERR2199847	ERR2199912	ERR2199965	ERR2200023	ERR2200089	ERR2200151	ERR2510146	ERR2510198

ERR2510199	ERR2510251	ERR2510302	ERR2510353	ERR2510404	ERR2510455	ERR2510506	ERR2510557
ERR2510200	ERR2510252	ERR2510303	ERR2510354	ERR2510405	ERR2510456	ERR2510507	ERR2510558
ERR2510201	ERR2510253	ERR2510304	ERR2510355	ERR2510406	ERR2510457	ERR2510508	ERR2510559
ERR2510202	ERR2510254	ERR2510305	ERR2510356	ERR2510407	ERR2510458	ERR2510509	ERR2510560
ERR2510203	ERR2510255	ERR2510306	ERR2510357	ERR2510408	ERR2510459	ERR2510510	ERR2510561
ERR2510204	ERR2510256	ERR2510307	ERR2510358	ERR2510409	ERR2510460	ERR2510511	ERR2510562
ERR2510205	ERR2510257	ERR2510308	ERR2510359	ERR2510410	ERR2510461	ERR2510512	ERR2510563
ERR2510207	ERR2510258	ERR2510309	ERR2510360	ERR2510411	ERR2510462	ERR2510513	ERR2510564
ERR2510208	ERR2510259	ERR2510310	ERR2510361	ERR2510412	ERR2510463	ERR2510514	ERR2510565
ERR2510209	ERR2510260	ERR2510311	ERR2510362	ERR2510413	ERR2510464	ERR2510515	ERR2510566
ERR2510210	ERR2510261	ERR2510312	ERR2510363	ERR2510414	ERR2510465	ERR2510516	ERR2510567
ERR2510211	ERR2510262	ERR2510313	ERR2510364	ERR2510415	ERR2510466	ERR2510517	ERR2510568
ERR2510212	ERR2510263	ERR2510314	ERR2510365	ERR2510416	ERR2510467	ERR2510518	ERR2510569
ERR2510213	ERR2510264	ERR2510315	ERR2510366	ERR2510417	ERR2510468	ERR2510519	ERR2510570
ERR2510214	ERR2510265	ERR2510316	ERR2510367	ERR2510418	ERR2510469	ERR2510520	ERR2510571
ERR2510215	ERR2510266	ERR2510317	ERR2510368	ERR2510419	ERR2510470	ERR2510521	ERR2510572
ERR2510216	ERR2510267	ERR2510318	ERR2510369	ERR2510420	ERR2510471	ERR2510522	ERR2510573
ERR2510217	ERR2510268	ERR2510319	ERR2510370	ERR2510421	ERR2510472	ERR2510523	ERR2510574
ERR2510218	ERR2510269	ERR2510320	ERR2510371	ERR2510422	ERR2510473	ERR2510524	ERR2510575
ERR2510219	ERR2510270	ERR2510321	ERR2510372	ERR2510423	ERR2510474	ERR2510525	ERR2510576
ERR2510220	ERR2510271	ERR2510322	ERR2510373	ERR2510424	ERR2510475	ERR2510526	ERR2510577
ERR2510221	ERR2510272	ERR2510323	ERR2510374	ERR2510425	ERR2510476	ERR2510527	ERR2510578
ERR2510222	ERR2510273	ERR2510324	ERR2510375	ERR2510426	ERR2510477	ERR2510528	ERR2510579
ERR2510223	ERR2510274	ERR2510325	ERR2510376	ERR2510427	ERR2510478	ERR2510529	ERR2510580
ERR2510224	ERR2510275	ERR2510326	ERR2510377	ERR2510428	ERR2510479	ERR2510530	ERR2510581
ERR2510225	ERR2510276	ERR2510327	ERR2510378	ERR2510429	ERR2510480	ERR2510531	ERR2510582
ERR2510226	ERR2510277	ERR2510328	ERR2510379	ERR2510430	ERR2510481	ERR2510532	ERR2510583
ERR2510227	ERR2510278	ERR2510329	ERR2510380	ERR2510431	ERR2510482	ERR2510533	ERR2510584
ERR2510228	ERR2510279	ERR2510330	ERR2510381	ERR2510432	ERR2510483	ERR2510534	ERR2510585
ERR2510229	ERR2510280	ERR2510331	ERR2510382	ERR2510433	ERR2510484	ERR2510535	ERR2510586
ERR2510230	ERR2510281	ERR2510332	ERR2510383	ERR2510434	ERR2510485	ERR2510536	ERR2510587
ERR2510231	ERR2510282	ERR2510333	ERR2510384	ERR2510435	ERR2510486	ERR2510537	ERR2510588
ERR2510232	ERR2510283	ERR2510334	ERR2510385	ERR2510436	ERR2510487	ERR2510538	ERR2510589
ERR2510233	ERR2510284	ERR2510335	ERR2510386	ERR2510437	ERR2510488	ERR2510539	ERR2510590
ERR2510234	ERR2510285	ERR2510336	ERR2510387	ERR2510438	ERR2510489	ERR2510540	ERR2510591
ERR2510235	ERR2510286	ERR2510337	ERR2510388	ERR2510439	ERR2510490	ERR2510541	ERR2510592
ERR2510236	ERR2510287	ERR2510338	ERR2510389	ERR2510440	ERR2510491	ERR2510542	ERR2510593
ERR2510237	ERR2510288	ERR2510339	ERR2510390	ERR2510441	ERR2510492	ERR2510543	ERR2510594
ERR2510238	ERR2510289	ERR2510340	ERR2510391	ERR2510442	ERR2510493	ERR2510544	ERR2510595
ERR2510239	ERR2510290	ERR2510341	ERR2510392	ERR2510443	ERR2510494	ERR2510545	ERR2510596
ERR2510240	ERR2510291	ERR2510342	ERR2510393	ERR2510444	ERR2510495	ERR2510546	ERR2510597
ERR2510241	ERR2510292	ERR2510343	ERR2510394	ERR2510445	ERR2510496	ERR2510547	ERR2510598
ERR2510242	ERR2510293	ERR2510344	ERR2510395	ERR2510446	ERR2510497	ERR2510548	ERR2510599
ERR2510243	ERR2510294	ERR2510345	ERR2510396	ERR2510447	ERR2510498	ERR2510549	ERR2510600
ERR2510244	ERR2510295	ERR2510346	ERR2510397	ERR2510448	ERR2510499	ERR2510550	ERR2510601
ERR2510245	ERR2510296	ERR2510347	ERR2510398	ERR2510449	ERR2510500	ERR2510551	ERR2510602
ERR2510246	ERR2510297	ERR2510348	ERR2510399	ERR2510450	ERR2510501	ERR2510552	ERR2510603
ERR2510247	ERR2510298	ERR2510349	ERR2510400	ERR2510451	ERR2510502	ERR2510553	ERR2510604
ERR2510248	ERR2510299	ERR2510350	ERR2510401	ERR2510452	ERR2510503	ERR2510554	ERR2510605
ERR2510249	ERR2510300	ERR2510351	ERR2510402	ERR2510453	ERR2510504	ERR2510555	ERR2510606
ERR2510250	ERR2510301	ERR2510352	ERR2510403	ERR2510454	ERR2510505	ERR2510556	ERR2510607

ERR2510608	ERR2510659	ERR2510710	ERR2510761	ERR2510812	ERR2510863	ERR2512417	ERR2512468
ERR2510609	ERR2510660	ERR2510711	ERR2510762	ERR2510813	ERR2510864	ERR2512418	ERR2512469
ERR2510610	ERR2510661	ERR2510712	ERR2510763	ERR2510814	ERR2510865	ERR2512419	ERR2512470
ERR2510611	ERR2510662	ERR2510713	ERR2510764	ERR2510815	ERR2510866	ERR2512420	ERR2512471
ERR2510612	ERR2510663	ERR2510714	ERR2510765	ERR2510816	ERR2510867	ERR2512421	ERR2512472
ERR2510613	ERR2510664	ERR2510715	ERR2510766	ERR2510817	ERR2510868	ERR2512422	ERR2512473
ERR2510614	ERR2510665	ERR2510716	ERR2510767	ERR2510818	ERR2510869	ERR2512423	ERR2512474
ERR2510615	ERR2510666	ERR2510717	ERR2510768	ERR2510819	ERR2510870	ERR2512424	ERR2512475
ERR2510616	ERR2510667	ERR2510718	ERR2510769	ERR2510820	ERR2510871	ERR2512425	ERR2512476
ERR2510617	ERR2510668	ERR2510719	ERR2510770	ERR2510821	ERR2510872	ERR2512426	ERR2512477
ERR2510618	ERR2510669	ERR2510720	ERR2510771	ERR2510822	ERR2510873	ERR2512427	ERR2512478
ERR2510619	ERR2510670	ERR2510721	ERR2510772	ERR2510823	ERR2510874	ERR2512428	ERR2512479
ERR2510620	ERR2510671	ERR2510722	ERR2510773	ERR2510824	ERR2510875	ERR2512429	ERR2512480
ERR2510621	ERR2510672	ERR2510723	ERR2510774	ERR2510825	ERR2510876	ERR2512430	ERR2512481
ERR2510622	ERR2510673	ERR2510724	ERR2510775	ERR2510826	ERR2510877	ERR2512431	ERR2512482
ERR2510623	ERR2510674	ERR2510725	ERR2510776	ERR2510827	ERR2510878	ERR2512432	ERR2512483
ERR2510624	ERR2510675	ERR2510726	ERR2510777	ERR2510828	ERR2510879	ERR2512433	ERR2512484
ERR2510625	ERR2510676	ERR2510727	ERR2510778	ERR2510829	ERR2510880	ERR2512434	ERR2512485
ERR2510626	ERR2510677	ERR2510728	ERR2510779	ERR2510830	ERR2510881	ERR2512435	ERR2512486
ERR2510627	ERR2510678	ERR2510729	ERR2510780	ERR2510831	ERR2510882	ERR2512436	ERR2512487
ERR2510628	ERR2510679	ERR2510730	ERR2510781	ERR2510832	ERR2510883	ERR2512437	ERR2512488
ERR2510629	ERR2510680	ERR2510731	ERR2510782	ERR2510833	ERR2512386	ERR2512438	ERR2512489
ERR2510630	ERR2510681	ERR2510732	ERR2510783	ERR2510834	ERR2512387	ERR2512439	ERR2512490
ERR2510631	ERR2510682	ERR2510733	ERR2510784	ERR2510835	ERR2512388	ERR2512440	ERR2512491
ERR2510632	ERR2510683	ERR2510734	ERR2510785	ERR2510836	ERR2512390	ERR2512441	ERR2512492
ERR2510633	ERR2510684	ERR2510735	ERR2510786	ERR2510837	ERR2512391	ERR2512442	ERR2512493
ERR2510634	ERR2510685	ERR2510736	ERR2510787	ERR2510838	ERR2512392	ERR2512443	ERR2512494
ERR2510635	ERR2510686	ERR2510737	ERR2510788	ERR2510839	ERR2512393	ERR2512444	ERR2512496
ERR2510636	ERR2510687	ERR2510738	ERR2510789	ERR2510840	ERR2512394	ERR2512445	ERR2512497
ERR2510637	ERR2510688	ERR2510739	ERR2510790	ERR2510841	ERR2512395	ERR2512446	ERR2512498
ERR2510638	ERR2510689	ERR2510740	ERR2510791	ERR2510842	ERR2512396	ERR2512447	ERR2512499
ERR2510639	ERR2510690	ERR2510741	ERR2510792	ERR2510843	ERR2512397	ERR2512448	ERR2512500
ERR2510640	ERR2510691	ERR2510742	ERR2510793	ERR2510844	ERR2512398	ERR2512449	ERR2512501
ERR2510641	ERR2510692	ERR2510743	ERR2510794	ERR2510845	ERR2512399	ERR2512450	ERR2512502
ERR2510642	ERR2510693	ERR2510744	ERR2510795	ERR2510846	ERR2512400	ERR2512451	ERR2512503
ERR2510643	ERR2510694	ERR2510745	ERR2510796	ERR2510847	ERR2512401	ERR2512452	ERR2512504
ERR2510644	ERR2510695	ERR2510746	ERR2510797	ERR2510848	ERR2512402	ERR2512453	ERR2512505
ERR2510645	ERR2510696	ERR2510747	ERR2510798	ERR2510849	ERR2512403	ERR2512454	ERR2512506
ERR2510646	ERR2510697	ERR2510748	ERR2510799	ERR2510850	ERR2512404	ERR2512455	ERR2512507
ERR2510647	ERR2510698	ERR2510749	ERR2510800	ERR2510851	ERR2512405	ERR2512456	ERR2512508
ERR2510648	ERR2510699	ERR2510750	ERR2510801	ERR2510852	ERR2512406	ERR2512457	ERR2512509
ERR2510649	ERR2510700	ERR2510751	ERR2510802	ERR2510853	ERR2512407	ERR2512458	ERR2512510
ERR2510650	ERR2510701	ERR2510752	ERR2510803	ERR2510854	ERR2512408	ERR2512459	ERR2512511
ERR2510651	ERR2510702	ERR2510753	ERR2510804	ERR2510855	ERR2512409	ERR2512460	ERR2512512
ERR2510652	ERR2510703	ERR2510754	ERR2510805	ERR2510856	ERR2512410	ERR2512461	ERR2512513
ERR2510653	ERR2510704	ERR2510755	ERR2510806	ERR2510857	ERR2512411	ERR2512462	ERR2512514
ERR2510654	ERR2510705	ERR2510756	ERR2510807	ERR2510858	ERR2512412	ERR2512463	ERR2512515
ERR2510655	ERR2510706	ERR2510757	ERR2510808	ERR2510859	ERR2512413	ERR2512464	ERR2512516
ERR2510656	ERR2510707	ERR2510758	ERR2510809	ERR2510860	ERR2512414	ERR2512465	ERR2512517
ERR2510657	ERR2510708	ERR2510759	ERR2510810	ERR2510861	ERR2512415	ERR2512466	ERR2512518
ERR2510658	ERR2510709	ERR2510760	ERR2510811	ERR2510862	ERR2512416	ERR2512467	ERR2512519

ERR2512520	ERR2512574	ERR2512625	ERR2512679	ERR2512730	ERR2512783	ERR2512836	ERR2512890
ERR2512521	ERR2512575	ERR2512626	ERR2512680	ERR2512731	ERR2512784	ERR2512837	ERR2512891
ERR2512522	ERR2512576	ERR2512627	ERR2512681	ERR2512733	ERR2512785	ERR2512840	ERR2512892
ERR2512523	ERR2512577	ERR2512628	ERR2512682	ERR2512734	ERR2512786	ERR2512841	ERR2512893
ERR2512524	ERR2512578	ERR2512629	ERR2512683	ERR2512735	ERR2512787	ERR2512842	ERR2512894
ERR2512525	ERR2512579	ERR2512630	ERR2512684	ERR2512736	ERR2512789	ERR2512843	ERR2512895
ERR2512526	ERR2512580	ERR2512631	ERR2512685	ERR2512737	ERR2512790	ERR2512844	ERR2512896
ERR2512527	ERR2512581	ERR2512632	ERR2512686	ERR2512738	ERR2512791	ERR2512845	ERR2512897
ERR2512528	ERR2512582	ERR2512633	ERR2512687	ERR2512739	ERR2512792	ERR2512846	ERR2512898
ERR2512529	ERR2512583	ERR2512634	ERR2512688	ERR2512740	ERR2512793	ERR2512847	ERR2512899
ERR2512530	ERR2512584	ERR2512635	ERR2512689	ERR2512741	ERR2512794	ERR2512848	ERR2512900
ERR2512531	ERR2512585	ERR2512636	ERR2512690	ERR2512742	ERR2512795	ERR2512849	ERR2512901
ERR2512532	ERR2512586	ERR2512637	ERR2512691	ERR2512743	ERR2512796	ERR2512850	ERR2512902
ERR2512534	ERR2512587	ERR2512638	ERR2512692	ERR2512744	ERR2512797	ERR2512851	ERR2512903
ERR2512535	ERR2512588	ERR2512639	ERR2512693	ERR2512745	ERR2512798	ERR2512852	ERR2512904
ERR2512536	ERR2512589	ERR2512640	ERR2512694	ERR2512746	ERR2512799	ERR2512853	ERR2512905
ERR2512537	ERR2512590	ERR2512641	ERR2512695	ERR2512747	ERR2512800	ERR2512854	ERR2512906
ERR2512538	ERR2512591	ERR2512642	ERR2512696	ERR2512748	ERR2512801	ERR2512855	ERR2512907
ERR2512539	ERR2512592	ERR2512643	ERR2512697	ERR2512749	ERR2512802	ERR2512856	ERR2512908
ERR2512540	ERR2512593	ERR2512644	ERR2512698	ERR2512750	ERR2512803	ERR2512857	ERR2512909
ERR2512541	ERR2512594	ERR2512645	ERR2512699	ERR2512751	ERR2512804	ERR2512858	ERR2512910
ERR2512542	ERR2512595	ERR2512646	ERR2512700	ERR2512752	ERR2512805	ERR2512860	ERR2512911
ERR2512543	ERR2512596	ERR2512648	ERR2512701	ERR2512753	ERR2512806	ERR2512861	ERR2512912
ERR2512544	ERR2512597	ERR2512649	ERR2512702	ERR2512754	ERR2512807	ERR2512862	ERR2512913
ERR2512545	ERR2512598	ERR2512650	ERR2512703	ERR2512756	ERR2512808	ERR2512863	ERR2512914
ERR2512546	ERR2512599	ERR2512651	ERR2512704	ERR2512757	ERR2512809	ERR2512864	ERR2512915
ERR2512547	ERR2512600	ERR2512652	ERR2512705	ERR2512758	ERR2512810	ERR2512865	ERR2512916
ERR2512548	ERR2512601	ERR2512653	ERR2512706	ERR2512759	ERR2512811	ERR2512866	ERR2512917
ERR2512550	ERR2512602	ERR2512654	ERR2512707	ERR2512760	ERR2512812	ERR2512867	ERR2512918
ERR2512551	ERR2512603	ERR2512655	ERR2512708	ERR2512761	ERR2512813	ERR2512868	ERR2512919
ERR2512552	ERR2512604	ERR2512656	ERR2512709	ERR2512762	ERR2512814	ERR2512869	ERR2512920
ERR2512553	ERR2512605	ERR2512657	ERR2512710	ERR2512763	ERR2512815	ERR2512870	ERR2512922
ERR2512554	ERR2512606	ERR2512658	ERR2512711	ERR2512764	ERR2512816	ERR2512871	ERR2512923
ERR2512555	ERR2512607	ERR2512659	ERR2512712	ERR2512765	ERR2512817	ERR2512872	ERR2512924
ERR2512556	ERR2512608	ERR2512660	ERR2512713	ERR2512766	ERR2512818	ERR2512873	ERR2512925
ERR2512557	ERR2512609	ERR2512661	ERR2512714	ERR2512767	ERR2512819	ERR2512874	ERR2512926
ERR2512558	ERR2512610	ERR2512662	ERR2512715	ERR2512768	ERR2512820	ERR2512875	ERR2512927
ERR2512559	ERR2512611	ERR2512663	ERR2512716	ERR2512769	ERR2512821	ERR2512876	ERR2512928
ERR2512560	ERR2512612	ERR2512664	ERR2512717	ERR2512770	ERR2512822	ERR2512877	ERR2512929
ERR2512561	ERR2512613	ERR2512665	ERR2512718	ERR2512771	ERR2512823	ERR2512878	ERR2512930
ERR2512562	ERR2512614	ERR2512666	ERR2512719	ERR2512772	ERR2512824	ERR2512879	ERR2512932
ERR2512563	ERR2512615	ERR2512667	ERR2512720	ERR2512773	ERR2512825	ERR2512880	ERR2512933
ERR2512564	ERR2512616	ERR2512668	ERR2512721	ERR2512774	ERR2512827	ERR2512881	ERR2512934
ERR2512565	ERR2512617	ERR2512669	ERR2512722	ERR2512775	ERR2512828	ERR2512882	ERR2512935
ERR2512566	ERR2512618	ERR2512670	ERR2512723	ERR2512776	ERR2512829	ERR2512883	ERR2512936
ERR2512567	ERR2512619	ERR2512671	ERR2512724	ERR2512777	ERR2512830	ERR2512884	ERR2512937
ERR2512568	ERR2512620	ERR2512672	ERR2512725	ERR2512778	ERR2512831	ERR2512885	ERR2512938
ERR2512569	ERR2512621	ERR2512673	ERR2512726	ERR2512779	ERR2512832	ERR2512886	ERR2512939
ERR2512571	ERR2512622	ERR2512674	ERR2512727	ERR2512780	ERR2512833	ERR2512887	ERR2512940
ERR2512572	ERR2512623	ERR2512677	ERR2512728	ERR2512781	ERR2512834	ERR2512888	ERR2512941
ERR2512573	ERR2512624	ERR2512678	ERR2512729	ERR2512782	ERR2512835	ERR2512889	ERR2512943

ERR2512944	ERR2512997	ERR2513049	ERR2513100	ERR2513152	ERR2513205	ERR2513256	ERR2513308
ERR2512945	ERR2512998	ERR2513050	ERR2513101	ERR2513153	ERR2513206	ERR2513257	ERR2513309
ERR2512946	ERR2512999	ERR2513051	ERR2513102	ERR2513154	ERR2513207	ERR2513258	ERR2513310
ERR2512947	ERR2513000	ERR2513052	ERR2513103	ERR2513155	ERR2513208	ERR2513259	ERR2513311
ERR2512948	ERR2513001	ERR2513053	ERR2513104	ERR2513156	ERR2513209	ERR2513260	ERR2513312
ERR2512949	ERR2513002	ERR2513054	ERR2513105	ERR2513157	ERR2513210	ERR2513261	ERR2513313
ERR2512950	ERR2513003	ERR2513055	ERR2513106	ERR2513158	ERR2513211	ERR2513262	ERR2513314
ERR2512951	ERR2513004	ERR2513056	ERR2513107	ERR2513159	ERR2513212	ERR2513263	ERR2513315
ERR2512952	ERR2513005	ERR2513057	ERR2513108	ERR2513160	ERR2513213	ERR2513264	ERR2513316
ERR2512953	ERR2513006	ERR2513058	ERR2513109	ERR2513161	ERR2513214	ERR2513265	ERR2513317
ERR2512955	ERR2513007	ERR2513059	ERR2513110	ERR2513162	ERR2513215	ERR2513266	ERR2513318
ERR2512956	ERR2513008	ERR2513060	ERR2513111	ERR2513163	ERR2513216	ERR2513267	ERR2513319
ERR2512957	ERR2513009	ERR2513061	ERR2513112	ERR2513164	ERR2513217	ERR2513268	ERR2513320
ERR2512958	ERR2513010	ERR2513062	ERR2513113	ERR2513165	ERR2513218	ERR2513269	ERR2513321
ERR2512959	ERR2513011	ERR2513063	ERR2513114	ERR2513166	ERR2513219	ERR2513270	ERR2513322
ERR2512960	ERR2513012	ERR2513064	ERR2513115	ERR2513167	ERR2513220	ERR2513271	ERR2513323
ERR2512961	ERR2513013	ERR2513065	ERR2513116	ERR2513168	ERR2513221	ERR2513272	ERR2513324
ERR2512963	ERR2513014	ERR2513066	ERR2513117	ERR2513169	ERR2513222	ERR2513273	ERR2513325
ERR2512964	ERR2513015	ERR2513067	ERR2513118	ERR2513170	ERR2513223	ERR2513274	ERR2513326
ERR2512965	ERR2513016	ERR2513068	ERR2513119	ERR2513171	ERR2513224	ERR2513275	ERR2513327
ERR2512966	ERR2513017	ERR2513069	ERR2513120	ERR2513172	ERR2513225	ERR2513276	ERR2513328
ERR2512967	ERR2513018	ERR2513070	ERR2513121	ERR2513173	ERR2513226	ERR2513277	ERR2513329
ERR2512968	ERR2513019	ERR2513071	ERR2513122	ERR2513174	ERR2513227	ERR2513278	ERR2513330
ERR2512969	ERR2513020	ERR2513072	ERR2513123	ERR2513175	ERR2513228	ERR2513279	ERR2513331
ERR2512970	ERR2513021	ERR2513073	ERR2513124	ERR2513176	ERR2513229	ERR2513280	ERR2513332
ERR2512971	ERR2513022	ERR2513074	ERR2513125	ERR2513177	ERR2513230	ERR2513281	ERR2513333
ERR2512972	ERR2513023	ERR2513075	ERR2513126	ERR2513178	ERR2513231	ERR2513282	ERR2513334
ERR2512973	ERR2513024	ERR2513076	ERR2513127	ERR2513179	ERR2513232	ERR2513283	ERR2513335
ERR2512974	ERR2513025	ERR2513077	ERR2513128	ERR2513180	ERR2513233	ERR2513284	ERR2513336
ERR2512975	ERR2513026	ERR2513078	ERR2513129	ERR2513181	ERR2513234	ERR2513285	ERR2513337
ERR2512976	ERR2513027	ERR2513079	ERR2513130	ERR2513182	ERR2513235	ERR2513286	ERR2513338
ERR2512977	ERR2513028	ERR2513080	ERR2513131	ERR2513183	ERR2513236	ERR2513287	ERR2513339
ERR2512978	ERR2513029	ERR2513081	ERR2513132	ERR2513184	ERR2513237	ERR2513288	ERR2513340
ERR2512979	ERR2513030	ERR2513082	ERR2513133	ERR2513185	ERR2513238	ERR2513289	ERR2513341
ERR2512980	ERR2513031	ERR2513083	ERR2513134	ERR2513186	ERR2513239	ERR2513290	ERR2513342
ERR2512981	ERR2513032	ERR2513084	ERR2513135	ERR2513187	ERR2513240	ERR2513291	ERR2513343
ERR2512982	ERR2513033	ERR2513085	ERR2513136	ERR2513188	ERR2513241	ERR2513292	ERR2513344
ERR2512983	ERR2513035	ERR2513086	ERR2513137	ERR2513189	ERR2513242	ERR2513293	ERR2513345
ERR2512984	ERR2513036	ERR2513087	ERR2513139	ERR2513190	ERR2513243	ERR2513294	ERR2513346
ERR2512985	ERR2513037	ERR2513088	ERR2513140	ERR2513191	ERR2513244	ERR2513295	ERR2513347
ERR2512986	ERR2513038	ERR2513089	ERR2513141	ERR2513192	ERR2513245	ERR2513296	ERR2513348
ERR2512987	ERR2513039	ERR2513090	ERR2513142	ERR2513193	ERR2513246	ERR2513297	ERR2513349
ERR2512988	ERR2513040	ERR2513091	ERR2513143	ERR2513196	ERR2513247	ERR2513298	ERR2513350
ERR2512989	ERR2513041	ERR2513092	ERR2513144	ERR2513197	ERR2513248	ERR2513299	ERR2513351
ERR2512990	ERR2513042	ERR2513093	ERR2513145	ERR2513198	ERR2513249	ERR2513300	ERR2513352
ERR2512991	ERR2513043	ERR2513094	ERR2513146	ERR2513199	ERR2513250	ERR2513301	ERR2513353
ERR2512992	ERR2513044	ERR2513095	ERR2513147	ERR2513200	ERR2513251	ERR2513303	ERR2513354
ERR2512993	ERR2513045	ERR2513096	ERR2513148	ERR2513201	ERR2513252	ERR2513304	ERR2513355
ERR2512994	ERR2513046	ERR2513097	ERR2513149	ERR2513202	ERR2513253	ERR2513305	ERR2513356
ERR2512995	ERR2513047	ERR2513098	ERR2513150	ERR2513203	ERR2513254	ERR2513306	ERR2513357
ERR2512996	ERR2513048	ERR2513099	ERR2513151	ERR2513204	ERR2513255	ERR2513307	ERR2513358

ERR2513359	ERR2513410	ERR2513463	ERR2513514	ERR2513565	ERR2513617	ERR2513669	ERR2513721
ERR2513360	ERR2513411	ERR2513464	ERR2513515	ERR2513566	ERR2513618	ERR2513670	ERR2513722
ERR2513361	ERR2513412	ERR2513465	ERR2513516	ERR2513567	ERR2513619	ERR2513672	ERR2513723
ERR2513362	ERR2513413	ERR2513466	ERR2513517	ERR2513568	ERR2513620	ERR2513673	ERR2513724
ERR2513363	ERR2513414	ERR2513467	ERR2513518	ERR2513569	ERR2513621	ERR2513674	ERR2513725
ERR2513364	ERR2513415	ERR2513468	ERR2513519	ERR2513570	ERR2513622	ERR2513675	ERR2513726
ERR2513365	ERR2513416	ERR2513469	ERR2513520	ERR2513571	ERR2513623	ERR2513676	ERR2513727
ERR2513366	ERR2513417	ERR2513470	ERR2513521	ERR2513572	ERR2513624	ERR2513677	ERR2513728
ERR2513367	ERR2513418	ERR2513471	ERR2513522	ERR2513573	ERR2513626	ERR2513678	ERR2513729
ERR2513368	ERR2513419	ERR2513472	ERR2513523	ERR2513574	ERR2513627	ERR2513679	ERR2513730
ERR2513369	ERR2513420	ERR2513473	ERR2513524	ERR2513575	ERR2513628	ERR2513680	ERR2513731
ERR2513370	ERR2513421	ERR2513474	ERR2513525	ERR2513576	ERR2513629	ERR2513681	ERR2513732
ERR2513371	ERR2513422	ERR2513475	ERR2513526	ERR2513577	ERR2513630	ERR2513682	ERR2513733
ERR2513372	ERR2513423	ERR2513476	ERR2513527	ERR2513578	ERR2513631	ERR2513683	ERR2513734
ERR2513373	ERR2513424	ERR2513477	ERR2513528	ERR2513579	ERR2513632	ERR2513684	ERR2513735
ERR2513374	ERR2513425	ERR2513478	ERR2513529	ERR2513580	ERR2513633	ERR2513685	ERR2513736
ERR2513375	ERR2513426	ERR2513479	ERR2513530	ERR2513581	ERR2513634	ERR2513686	ERR2513737
ERR2513376	ERR2513427	ERR2513480	ERR2513531	ERR2513582	ERR2513635	ERR2513687	ERR2513738
ERR2513377	ERR2513429	ERR2513481	ERR2513532	ERR2513583	ERR2513636	ERR2513688	ERR2513739
ERR2513378	ERR2513430	ERR2513482	ERR2513533	ERR2513584	ERR2513637	ERR2513689	ERR2513740
ERR2513379	ERR2513431	ERR2513483	ERR2513534	ERR2513585	ERR2513638	ERR2513690	ERR2513741
ERR2513380	ERR2513432	ERR2513484	ERR2513535	ERR2513586	ERR2513639	ERR2513691	ERR2513742
ERR2513381	ERR2513434	ERR2513485	ERR2513536	ERR2513587	ERR2513640	ERR2513692	ERR2513743
ERR2513382	ERR2513435	ERR2513486	ERR2513537	ERR2513588	ERR2513641	ERR2513693	ERR2513744
ERR2513383	ERR2513436	ERR2513487	ERR2513538	ERR2513589	ERR2513642	ERR2513694	ERR2513745
ERR2513384	ERR2513437	ERR2513488	ERR2513539	ERR2513590	ERR2513643	ERR2513695	ERR2513746
ERR2513385	ERR2513438	ERR2513489	ERR2513540	ERR2513591	ERR2513644	ERR2513696	ERR2513747
ERR2513386	ERR2513439	ERR2513490	ERR2513541	ERR2513592	ERR2513645	ERR2513697	ERR2513748
ERR2513387	ERR2513440	ERR2513491	ERR2513542	ERR2513593	ERR2513646	ERR2513698	ERR2513749
ERR2513388	ERR2513441	ERR2513492	ERR2513543	ERR2513594	ERR2513647	ERR2513699	ERR2513750
ERR2513389	ERR2513442	ERR2513493	ERR2513544	ERR2513595	ERR2513648	ERR2513700	ERR2513751
ERR2513390	ERR2513443	ERR2513494	ERR2513545	ERR2513596	ERR2513649	ERR2513701	ERR2513752
ERR2513391	ERR2513444	ERR2513495	ERR2513546	ERR2513597	ERR2513650	ERR2513702	ERR2513753
ERR2513392	ERR2513445	ERR2513496	ERR2513547	ERR2513598	ERR2513651	ERR2513703	ERR2513754
ERR2513393	ERR2513446	ERR2513497	ERR2513548	ERR2513599	ERR2513652	ERR2513704	ERR2513755
ERR2513394	ERR2513447	ERR2513498	ERR2513549	ERR2513600	ERR2513653	ERR2513705	ERR2513756
ERR2513395	ERR2513448	ERR2513499	ERR2513550	ERR2513601	ERR2513654	ERR2513706	ERR2513757
ERR2513396	ERR2513449	ERR2513500	ERR2513551	ERR2513602	ERR2513655	ERR2513707	ERR2513758
ERR2513397	ERR2513450	ERR2513501	ERR2513552	ERR2513603	ERR2513656	ERR2513708	ERR2513759
ERR2513398	ERR2513451	ERR2513502	ERR2513553	ERR2513604	ERR2513657	ERR2513709	ERR2513760
ERR2513399	ERR2513452	ERR2513503	ERR2513554	ERR2513605	ERR2513658	ERR2513710	ERR2513761
ERR2513400	ERR2513453	ERR2513504	ERR2513555	ERR2513606	ERR2513659	ERR2513711	ERR2513762
ERR2513401	ERR2513454	ERR2513505	ERR2513556	ERR2513608	ERR2513660	ERR2513712	ERR2513763
ERR2513402	ERR2513455	ERR2513506	ERR2513557	ERR2513609	ERR2513661	ERR2513713	ERR2513764
ERR2513403	ERR2513456	ERR2513507	ERR2513558	ERR2513610	ERR2513662	ERR2513714	ERR2513765
ERR2513404	ERR2513457	ERR2513508	ERR2513559	ERR2513611	ERR2513663	ERR2513715	ERR2513766
ERR2513405	ERR2513458	ERR2513509	ERR2513560	ERR2513612	ERR2513664	ERR2513716	ERR2513767
ERR2513406	ERR2513459	ERR2513510	ERR2513561	ERR2513613	ERR2513665	ERR2513717	ERR2513768
ERR2513407	ERR2513460	ERR2513511	ERR2513562	ERR2513614	ERR2513666	ERR2513718	ERR2513769
ERR2513408	ERR2513461	ERR2513512	ERR2513563	ERR2513615	ERR2513667	ERR2513719	ERR2513770
ERR2513409	ERR2513462	ERR2513513	ERR2513564	ERR2513616	ERR2513668	ERR2513720	ERR2513771

ERR2513772	ERR2513824	ERR2513875	ERR2513926	ERR2513977	ERR2514028	ERR2514079	ERR2514131
ERR2513773	ERR2513825	ERR2513876	ERR2513927	ERR2513978	ERR2514029	ERR2514080	ERR2514132
ERR2513774	ERR2513826	ERR2513877	ERR2513928	ERR2513979	ERR2514030	ERR2514081	ERR2514133
ERR2513775	ERR2513827	ERR2513878	ERR2513929	ERR2513980	ERR2514031	ERR2514082	ERR2514134
ERR2513776	ERR2513828	ERR2513879	ERR2513930	ERR2513981	ERR2514032	ERR2514083	ERR2514135
ERR2513777	ERR2513829	ERR2513880	ERR2513931	ERR2513982	ERR2514033	ERR2514084	ERR2514136
ERR2513778	ERR2513830	ERR2513881	ERR2513932	ERR2513983	ERR2514034	ERR2514085	ERR2514137
ERR2513779	ERR2513831	ERR2513882	ERR2513933	ERR2513984	ERR2514035	ERR2514086	ERR2514138
ERR2513780	ERR2513832	ERR2513883	ERR2513934	ERR2513985	ERR2514036	ERR2514088	ERR2514139
ERR2513781	ERR2513833	ERR2513884	ERR2513935	ERR2513986	ERR2514037	ERR2514089	ERR2514140
ERR2513782	ERR2513834	ERR2513885	ERR2513936	ERR2513987	ERR2514038	ERR2514090	ERR2514141
ERR2513783	ERR2513835	ERR2513886	ERR2513937	ERR2513988	ERR2514039	ERR2514091	ERR2514142
ERR2513784	ERR2513836	ERR2513887	ERR2513938	ERR2513989	ERR2514040	ERR2514092	ERR2514143
ERR2513785	ERR2513837	ERR2513888	ERR2513939	ERR2513990	ERR2514041	ERR2514093	ERR2514145
ERR2513786	ERR2513838	ERR2513889	ERR2513940	ERR2513991	ERR2514042	ERR2514094	ERR2514146
ERR2513787	ERR2513839	ERR2513890	ERR2513941	ERR2513992	ERR2514043	ERR2514095	ERR2514147
ERR2513788	ERR2513840	ERR2513891	ERR2513942	ERR2513993	ERR2514044	ERR2514096	ERR2514148
ERR2513789	ERR2513841	ERR2513892	ERR2513943	ERR2513994	ERR2514045	ERR2514097	ERR2514149
ERR2513790	ERR2513842	ERR2513893	ERR2513944	ERR2513995	ERR2514046	ERR2514098	ERR2514150
ERR2513791	ERR2513843	ERR2513894	ERR2513945	ERR2513996	ERR2514047	ERR2514099	ERR2514151
ERR2513792	ERR2513844	ERR2513895	ERR2513946	ERR2513997	ERR2514048	ERR2514100	ERR2514152
ERR2513793	ERR2513845	ERR2513896	ERR2513947	ERR2513998	ERR2514049	ERR2514101	ERR2514153
ERR2513794	ERR2513846	ERR2513897	ERR2513948	ERR2513999	ERR2514050	ERR2514102	ERR2514154
ERR2513795	ERR2513847	ERR2513898	ERR2513949	ERR2514000	ERR2514051	ERR2514103	ERR2514155
ERR2513796	ERR2513848	ERR2513899	ERR2513950	ERR2514001	ERR2514052	ERR2514104	ERR2514156
ERR2513797	ERR2513849	ERR2513900	ERR2513951	ERR2514002	ERR2514053	ERR2514105	ERR2514157
ERR2513798	ERR2513850	ERR2513901	ERR2513952	ERR2514003	ERR2514054	ERR2514106	ERR2514158
ERR2513799	ERR2513851	ERR2513902	ERR2513953	ERR2514004	ERR2514055	ERR2514107	ERR2514159
ERR2513800	ERR2513852	ERR2513903	ERR2513954	ERR2514005	ERR2514056	ERR2514108	ERR2514160
ERR2513801	ERR2513853	ERR2513904	ERR2513955	ERR2514006	ERR2514057	ERR2514109	ERR2514161
ERR2513802	ERR2513854	ERR2513905	ERR2513956	ERR2514007	ERR2514058	ERR2514110	ERR2514162
ERR2513803	ERR2513855	ERR2513906	ERR2513957	ERR2514008	ERR2514059	ERR2514111	ERR2514163
ERR2513804	ERR2513856	ERR2513907	ERR2513958	ERR2514009	ERR2514060	ERR2514112	ERR2514164
ERR2513805	ERR2513857	ERR2513908	ERR2513959	ERR2514010	ERR2514061	ERR2514113	ERR2514165
ERR2513806	ERR2513858	ERR2513909	ERR2513960	ERR2514011	ERR2514062	ERR2514114	ERR2514166
ERR2513807	ERR2513859	ERR2513910	ERR2513961	ERR2514012	ERR2514063	ERR2514115	ERR2514167
ERR2513808	ERR2513860	ERR2513911	ERR2513962	ERR2514013	ERR2514064	ERR2514116	ERR2514168
ERR2513809	ERR2513861	ERR2513912	ERR2513963	ERR2514014	ERR2514065	ERR2514117	ERR2514169
ERR2513810	ERR2513862	ERR2513913	ERR2513964	ERR2514015	ERR2514066	ERR2514118	ERR2514170
ERR2513812	ERR2513863	ERR2513914	ERR2513965	ERR2514016	ERR2514067	ERR2514119	ERR2514171
ERR2513813	ERR2513864	ERR2513915	ERR2513966	ERR2514017	ERR2514068	ERR2514120	ERR2514173
ERR2513814	ERR2513865	ERR2513916	ERR2513967	ERR2514018	ERR2514069	ERR2514121	ERR2514174
ERR2513815	ERR2513866	ERR2513917	ERR2513968	ERR2514019	ERR2514070	ERR2514122	ERR2514175
ERR2513816	ERR2513867	ERR2513918	ERR2513969	ERR2514020	ERR2514071	ERR2514123	ERR2514176
ERR2513817	ERR2513868	ERR2513919	ERR2513970	ERR2514021	ERR2514072	ERR2514124	ERR2514177
ERR2513818	ERR2513869	ERR2513920	ERR2513971	ERR2514022	ERR2514073	ERR2514125	ERR2514179
ERR2513819	ERR2513870	ERR2513921	ERR2513972	ERR2514023	ERR2514074	ERR2514126	ERR2514180
ERR2513820	ERR2513871	ERR2513922	ERR2513973	ERR2514024	ERR2514075	ERR2514127	ERR2514181
ERR2513821	ERR2513872	ERR2513923	ERR2513974	ERR2514025	ERR2514076	ERR2514128	ERR2514182
ERR2513822	ERR2513873	ERR2513924	ERR2513975	ERR2514026	ERR2514077	ERR2514129	ERR2514183
ERR2513823	ERR2513874	ERR2513925	ERR2513976	ERR2514027	ERR2514078	ERR2514130	ERR2514184

ERR2514185	ERR2514238	ERR2514290	ERR2514341	ERR2514393	ERR2514444	ERR2514495	ERR2514547
ERR2514186	ERR2514239	ERR2514291	ERR2514342	ERR2514394	ERR2514445	ERR2514496	ERR2514548
ERR2514187	ERR2514240	ERR2514292	ERR2514343	ERR2514395	ERR2514446	ERR2514497	ERR2514549
ERR2514188	ERR2514241	ERR2514293	ERR2514344	ERR2514396	ERR2514447	ERR2514498	ERR2514550
ERR2514189	ERR2514242	ERR2514294	ERR2514345	ERR2514397	ERR2514448	ERR2514499	ERR2514551
ERR2514190	ERR2514243	ERR2514295	ERR2514346	ERR2514398	ERR2514449	ERR2514500	ERR2514552
ERR2514192	ERR2514244	ERR2514296	ERR2514347	ERR2514399	ERR2514450	ERR2514501	ERR2514553
ERR2514194	ERR2514245	ERR2514297	ERR2514348	ERR2514400	ERR2514451	ERR2514502	ERR2514554
ERR2514195	ERR2514246	ERR2514298	ERR2514349	ERR2514401	ERR2514452	ERR2514503	ERR2514555
ERR2514196	ERR2514247	ERR2514299	ERR2514350	ERR2514402	ERR2514453	ERR2514504	ERR2514556
ERR2514197	ERR2514248	ERR2514300	ERR2514351	ERR2514403	ERR2514454	ERR2514505	ERR2514557
ERR2514198	ERR2514249	ERR2514301	ERR2514353	ERR2514404	ERR2514455	ERR2514506	ERR2514558
ERR2514199	ERR2514250	ERR2514302	ERR2514354	ERR2514405	ERR2514456	ERR2514507	ERR2514559
ERR2514200	ERR2514251	ERR2514303	ERR2514355	ERR2514406	ERR2514457	ERR2514508	ERR2514560
ERR2514201	ERR2514252	ERR2514304	ERR2514356	ERR2514407	ERR2514458	ERR2514509	ERR2514561
ERR2514202	ERR2514253	ERR2514305	ERR2514357	ERR2514408	ERR2514459	ERR2514510	ERR2514562
ERR2514203	ERR2514254	ERR2514306	ERR2514358	ERR2514409	ERR2514460	ERR2514511	ERR2514563
ERR2514204	ERR2514255	ERR2514307	ERR2514359	ERR2514410	ERR2514461	ERR2514512	ERR2514564
ERR2514205	ERR2514256	ERR2514308	ERR2514360	ERR2514411	ERR2514462	ERR2514513	ERR2514565
ERR2514206	ERR2514257	ERR2514309	ERR2514361	ERR2514412	ERR2514463	ERR2514514	ERR2514566
ERR2514207	ERR2514258	ERR2514310	ERR2514362	ERR2514413	ERR2514464	ERR2514515	ERR2514567
ERR2514208	ERR2514259	ERR2514311	ERR2514363	ERR2514414	ERR2514465	ERR2514516	ERR2514568
ERR2514209	ERR2514260	ERR2514312	ERR2514364	ERR2514415	ERR2514466	ERR2514517	ERR2514569
ERR2514210	ERR2514261	ERR2514313	ERR2514365	ERR2514416	ERR2514467	ERR2514518	ERR2514570
ERR2514211	ERR2514262	ERR2514314	ERR2514366	ERR2514417	ERR2514468	ERR2514519	ERR2514571
ERR2514212	ERR2514263	ERR2514315	ERR2514367	ERR2514418	ERR2514469	ERR2514520	ERR2514572
ERR2514213	ERR2514264	ERR2514316	ERR2514368	ERR2514419	ERR2514470	ERR2514521	ERR2514573
ERR2514214	ERR2514265	ERR2514317	ERR2514369	ERR2514420	ERR2514471	ERR2514522	ERR2514574
ERR2514215	ERR2514266	ERR2514318	ERR2514370	ERR2514421	ERR2514472	ERR2514523	ERR2514575
ERR2514216	ERR2514267	ERR2514319	ERR2514371	ERR2514422	ERR2514473	ERR2514524	ERR2514576
ERR2514217	ERR2514268	ERR2514320	ERR2514372	ERR2514423	ERR2514474	ERR2514525	ERR2514577
ERR2514218	ERR2514269	ERR2514321	ERR2514373	ERR2514424	ERR2514475	ERR2514526	ERR2514579
ERR2514219	ERR2514270	ERR2514322	ERR2514374	ERR2514425	ERR2514476	ERR2514527	ERR2514580
ERR2514220	ERR2514271	ERR2514323	ERR2514375	ERR2514426	ERR2514477	ERR2514528	ERR2514581
ERR2514221	ERR2514272	ERR2514324	ERR2514376	ERR2514427	ERR2514478	ERR2514529	ERR2514582
ERR2514222	ERR2514273	ERR2514325	ERR2514377	ERR2514428	ERR2514479	ERR2514530	ERR2514584
ERR2514223	ERR2514274	ERR2514326	ERR2514378	ERR2514429	ERR2514480	ERR2514531	ERR2514585
ERR2514224	ERR2514275	ERR2514327	ERR2514379	ERR2514430	ERR2514481	ERR2514532	ERR2514586
ERR2514225	ERR2514276	ERR2514328	ERR2514380	ERR2514431	ERR2514482	ERR2514533	ERR2514587
ERR2514226	ERR2514277	ERR2514329	ERR2514381	ERR2514432	ERR2514483	ERR2514534	ERR2514588
ERR2514227	ERR2514278	ERR2514330	ERR2514382	ERR2514433	ERR2514484	ERR2514535	ERR2514589
ERR2514228	ERR2514279	ERR2514331	ERR2514383	ERR2514434	ERR2514485	ERR2514536	ERR2514590
ERR2514229	ERR2514280	ERR2514332	ERR2514384	ERR2514435	ERR2514486	ERR2514537	ERR2514591
ERR2514230	ERR2514281	ERR2514333	ERR2514385	ERR2514436	ERR2514487	ERR2514538	ERR2514592
ERR2514231	ERR2514282	ERR2514334	ERR2514386	ERR2514437	ERR2514488	ERR2514539	ERR2514593
ERR2514232	ERR2514283	ERR2514335	ERR2514387	ERR2514438	ERR2514489	ERR2514540	ERR2514594
ERR2514233	ERR2514284	ERR2514336	ERR2514388	ERR2514439	ERR2514490	ERR2514541	ERR2514595
ERR2514234	ERR2514286	ERR2514337	ERR2514389	ERR2514440	ERR2514491	ERR2514542	ERR2514596
ERR2514235	ERR2514287	ERR2514338	ERR2514390	ERR2514441	ERR2514492	ERR2514543	ERR2514597
ERR2514236	ERR2514288	ERR2514339	ERR2514391	ERR2514442	ERR2514493	ERR2514544	ERR2514598
ERR2514237	ERR2514289	ERR2514340	ERR2514392	ERR2514443	ERR2514494	ERR2514546	ERR2514599

ERR2514600	ERR2514651	ERR2514702	ERR2514754	ERR2514805	ERR2514858	ERR2514909	ERR2514962
ERR2514601	ERR2514652	ERR2514703	ERR2514755	ERR2514806	ERR2514859	ERR2514910	ERR2514963
ERR2514602	ERR2514653	ERR2514704	ERR2514756	ERR2514807	ERR2514860	ERR2514911	ERR2514964
ERR2514603	ERR2514654	ERR2514705	ERR2514757	ERR2514808	ERR2514861	ERR2514912	ERR2514965
ERR2514604	ERR2514655	ERR2514706	ERR2514758	ERR2514809	ERR2514862	ERR2514913	ERR2514966
ERR2514605	ERR2514656	ERR2514707	ERR2514759	ERR2514810	ERR2514863	ERR2514914	ERR2514967
ERR2514606	ERR2514657	ERR2514708	ERR2514760	ERR2514811	ERR2514864	ERR2514915	ERR2514968
ERR2514607	ERR2514658	ERR2514709	ERR2514761	ERR2514812	ERR2514865	ERR2514916	ERR2514969
ERR2514608	ERR2514659	ERR2514710	ERR2514762	ERR2514813	ERR2514866	ERR2514917	ERR2514970
ERR2514609	ERR2514660	ERR2514711	ERR2514763	ERR2514814	ERR2514867	ERR2514918	ERR2514971
ERR2514610	ERR2514661	ERR2514712	ERR2514764	ERR2514815	ERR2514868	ERR2514919	ERR2514972
ERR2514611	ERR2514662	ERR2514713	ERR2514765	ERR2514816	ERR2514869	ERR2514920	ERR2514973
ERR2514612	ERR2514663	ERR2514714	ERR2514766	ERR2514817	ERR2514870	ERR2514921	ERR2514974
ERR2514613	ERR2514664	ERR2514715	ERR2514767	ERR2514818	ERR2514871	ERR2514922	ERR2514975
ERR2514614	ERR2514665	ERR2514716	ERR2514768	ERR2514819	ERR2514872	ERR2514923	ERR2514976
ERR2514615	ERR2514666	ERR2514717	ERR2514769	ERR2514820	ERR2514873	ERR2514924	ERR2514977
ERR2514616	ERR2514667	ERR2514718	ERR2514770	ERR2514821	ERR2514874	ERR2514925	ERR2514978
ERR2514617	ERR2514668	ERR2514719	ERR2514771	ERR2514822	ERR2514875	ERR2514926	ERR2514980
ERR2514618	ERR2514669	ERR2514720	ERR2514772	ERR2514823	ERR2514876	ERR2514927	ERR2514981
ERR2514619	ERR2514670	ERR2514721	ERR2514773	ERR2514824	ERR2514877	ERR2514928	ERR2514982
ERR2514620	ERR2514671	ERR2514722	ERR2514774	ERR2514825	ERR2514878	ERR2514929	ERR2514983
ERR2514621	ERR2514672	ERR2514723	ERR2514775	ERR2514826	ERR2514879	ERR2514930	ERR2514984
ERR2514622	ERR2514673	ERR2514724	ERR2514776	ERR2514827	ERR2514880	ERR2514931	ERR2514985
ERR2514623	ERR2514674	ERR2514725	ERR2514777	ERR2514828	ERR2514881	ERR2514932	ERR2514986
ERR2514624	ERR2514675	ERR2514726	ERR2514778	ERR2514829	ERR2514882	ERR2514933	ERR2514987
ERR2514625	ERR2514676	ERR2514727	ERR2514779	ERR2514830	ERR2514883	ERR2514934	ERR2514988
ERR2514626	ERR2514677	ERR2514728	ERR2514780	ERR2514831	ERR2514884	ERR2514935	ERR2514989
ERR2514627	ERR2514678	ERR2514729	ERR2514781	ERR2514833	ERR2514885	ERR2514936	ERR2514990
ERR2514628	ERR2514679	ERR2514730	ERR2514782	ERR2514834	ERR2514886	ERR2514937	ERR2514991
ERR2514629	ERR2514680	ERR2514731	ERR2514783	ERR2514835	ERR2514887	ERR2514938	ERR2514992
ERR2514630	ERR2514681	ERR2514732	ERR2514784	ERR2514836	ERR2514888	ERR2514939	ERR2514993
ERR2514631	ERR2514682	ERR2514733	ERR2514785	ERR2514838	ERR2514889	ERR2514940	ERR2514994
ERR2514632	ERR2514683	ERR2514734	ERR2514786	ERR2514839	ERR2514890	ERR2514941	ERR2514995
ERR2514633	ERR2514684	ERR2514735	ERR2514787	ERR2514840	ERR2514891	ERR2514942	ERR2514996
ERR2514634	ERR2514685	ERR2514736	ERR2514788	ERR2514841	ERR2514892	ERR2514943	ERR2514997
ERR2514635	ERR2514686	ERR2514737	ERR2514789	ERR2514842	ERR2514893	ERR2514944	ERR2514998
ERR2514636	ERR2514687	ERR2514739	ERR2514790	ERR2514843	ERR2514894	ERR2514945	ERR2514999
ERR2514637	ERR2514688	ERR2514740	ERR2514791	ERR2514844	ERR2514895	ERR2514946	ERR2515000
ERR2514638	ERR2514689	ERR2514741	ERR2514792	ERR2514845	ERR2514896	ERR2514947	ERR2515001
ERR2514639	ERR2514690	ERR2514742	ERR2514793	ERR2514846	ERR2514897	ERR2514948	ERR2515002
ERR2514640	ERR2514691	ERR2514743	ERR2514794	ERR2514847	ERR2514898	ERR2514949	ERR2515003
ERR2514641	ERR2514692	ERR2514744	ERR2514795	ERR2514848	ERR2514899	ERR2514950	ERR2515004
ERR2514642	ERR2514693	ERR2514745	ERR2514796	ERR2514849	ERR2514900	ERR2514951	ERR2515005
ERR2514643	ERR2514694	ERR2514746	ERR2514797	ERR2514850	ERR2514901	ERR2514952	ERR2515006
ERR2514644	ERR2514695	ERR2514747	ERR2514798	ERR2514851	ERR2514902	ERR2514953	ERR2515007
ERR2514645	ERR2514696	ERR2514748	ERR2514799	ERR2514852	ERR2514903	ERR2514954	ERR2515008
ERR2514646	ERR2514697	ERR2514749	ERR2514800	ERR2514853	ERR2514904	ERR2514956	ERR2515009
ERR2514647	ERR2514698	ERR2514750	ERR2514801	ERR2514854	ERR2514905	ERR2514958	ERR2515010
ERR2514648	ERR2514699	ERR2514751	ERR2514802	ERR2514855	ERR2514906	ERR2514959	ERR2515011
ERR2514649	ERR2514700	ERR2514752	ERR2514803	ERR2514856	ERR2514907	ERR2514960	ERR2515012
ERR2514650	ERR2514701	ERR2514753	ERR2514804	ERR2514857	ERR2514908	ERR2514961	ERR2515013

ERR2515014	ERR2515067	ERR2515121	ERR2515172	ERR2515223	ERR2515274	ERR2515325	ERR2515376
ERR2515015	ERR2515068	ERR2515122	ERR2515173	ERR2515224	ERR2515275	ERR2515326	ERR2515377
ERR2515017	ERR2515069	ERR2515123	ERR2515174	ERR2515225	ERR2515276	ERR2515327	ERR2515378
ERR2515018	ERR2515070	ERR2515124	ERR2515175	ERR2515226	ERR2515277	ERR2515328	ERR2515379
ERR2515019	ERR2515071	ERR2515125	ERR2515176	ERR2515227	ERR2515278	ERR2515329	ERR2515380
ERR2515020	ERR2515072	ERR2515126	ERR2515177	ERR2515228	ERR2515279	ERR2515330	ERR2515381
ERR2515021	ERR2515073	ERR2515127	ERR2515178	ERR2515229	ERR2515280	ERR2515331	ERR2515382
ERR2515022	ERR2515074	ERR2515128	ERR2515179	ERR2515230	ERR2515281	ERR2515332	ERR2515383
ERR2515023	ERR2515075	ERR2515129	ERR2515180	ERR2515231	ERR2515282	ERR2515333	ERR2515384
ERR2515024	ERR2515076	ERR2515130	ERR2515181	ERR2515232	ERR2515283	ERR2515334	ERR2515385
ERR2515025	ERR2515077	ERR2515131	ERR2515182	ERR2515233	ERR2515284	ERR2515335	ERR2515386
ERR2515026	ERR2515078	ERR2515132	ERR2515183	ERR2515234	ERR2515285	ERR2515336	ERR2515387
ERR2515027	ERR2515079	ERR2515133	ERR2515184	ERR2515235	ERR2515286	ERR2515337	ERR2515388
ERR2515028	ERR2515080	ERR2515134	ERR2515185	ERR2515236	ERR2515287	ERR2515338	ERR2515389
ERR2515029	ERR2515081	ERR2515135	ERR2515186	ERR2515237	ERR2515288	ERR2515339	ERR2515390
ERR2515030	ERR2515082	ERR2515136	ERR2515187	ERR2515238	ERR2515289	ERR2515340	ERR2515391
ERR2515031	ERR2515083	ERR2515137	ERR2515188	ERR2515239	ERR2515290	ERR2515341	ERR2515392
ERR2515032	ERR2515084	ERR2515138	ERR2515189	ERR2515240	ERR2515291	ERR2515342	ERR2515393
ERR2515033	ERR2515085	ERR2515139	ERR2515190	ERR2515241	ERR2515292	ERR2515343	ERR2515394
ERR2515034	ERR2515086	ERR2515140	ERR2515191	ERR2515242	ERR2515293	ERR2515344	ERR2515395
ERR2515035	ERR2515087	ERR2515141	ERR2515192	ERR2515243	ERR2515294	ERR2515345	ERR2515396
ERR2515036	ERR2515088	ERR2515142	ERR2515193	ERR2515244	ERR2515295	ERR2515346	ERR2515397
ERR2515037	ERR2515089	ERR2515143	ERR2515194	ERR2515245	ERR2515296	ERR2515347	ERR2515398
ERR2515038	ERR2515090	ERR2515144	ERR2515195	ERR2515246	ERR2515297	ERR2515348	ERR2515399
ERR2515039	ERR2515091	ERR2515145	ERR2515196	ERR2515247	ERR2515298	ERR2515349	ERR2515400
ERR2515040	ERR2515092	ERR2515146	ERR2515197	ERR2515248	ERR2515299	ERR2515350	ERR2515401
ERR2515041	ERR2515093	ERR2515147	ERR2515198	ERR2515249	ERR2515300	ERR2515351	ERR2515402
ERR2515042	ERR2515094	ERR2515148	ERR2515199	ERR2515250	ERR2515301	ERR2515352	ERR2515403
ERR2515043	ERR2515095	ERR2515149	ERR2515200	ERR2515251	ERR2515302	ERR2515353	ERR2515404
ERR2515044	ERR2515096	ERR2515150	ERR2515201	ERR2515252	ERR2515303	ERR2515354	ERR2515405
ERR2515045	ERR2515097	ERR2515151	ERR2515202	ERR2515253	ERR2515304	ERR2515355	ERR2515406
ERR2515046	ERR2515098	ERR2515152	ERR2515203	ERR2515254	ERR2515305	ERR2515356	ERR2515407
ERR2515047	ERR2515099	ERR2515153	ERR2515204	ERR2515255	ERR2515306	ERR2515357	ERR2515408
ERR2515048	ERR2515100	ERR2515154	ERR2515205	ERR2515256	ERR2515307	ERR2515358	ERR2515409
ERR2515049	ERR2515101	ERR2515155	ERR2515206	ERR2515257	ERR2515308	ERR2515359	ERR2515410
ERR2515050	ERR2515102	ERR2515156	ERR2515207	ERR2515258	ERR2515309	ERR2515360	ERR2515411
ERR2515052	ERR2515106	ERR2515157	ERR2515208	ERR2515259	ERR2515310	ERR2515361	ERR2515412
ERR2515053	ERR2515107	ERR2515158	ERR2515209	ERR2515260	ERR2515311	ERR2515362	ERR2515413
ERR2515054	ERR2515108	ERR2515159	ERR2515210	ERR2515261	ERR2515312	ERR2515363	ERR2515414
ERR2515055	ERR2515109	ERR2515160	ERR2515211	ERR2515262	ERR2515313	ERR2515364	ERR2515415
ERR2515056	ERR2515110	ERR2515161	ERR2515212	ERR2515263	ERR2515314	ERR2515365	ERR2515416
ERR2515057	ERR2515111	ERR2515162	ERR2515213	ERR2515264	ERR2515315	ERR2515366	ERR2515417
ERR2515058	ERR2515112	ERR2515163	ERR2515214	ERR2515265	ERR2515316	ERR2515367	ERR2515418
ERR2515059	ERR2515113	ERR2515164	ERR2515215	ERR2515266	ERR2515317	ERR2515368	ERR2515419
ERR2515060	ERR2515114	ERR2515165	ERR2515216	ERR2515267	ERR2515318	ERR2515369	ERR2515420
ERR2515061	ERR2515115	ERR2515166	ERR2515217	ERR2515268	ERR2515319	ERR2515370	ERR2515421
ERR2515062	ERR2515116	ERR2515167	ERR2515218	ERR2515269	ERR2515320	ERR2515371	ERR2515422
ERR2515063	ERR2515117	ERR2515168	ERR2515219	ERR2515270	ERR2515321	ERR2515372	ERR2515423
ERR2515064	ERR2515118	ERR2515169	ERR2515220	ERR2515271	ERR2515322	ERR2515373	ERR2515424
ERR2515065	ERR2515119	ERR2515170	ERR2515221	ERR2515272	ERR2515323	ERR2515374	ERR2515425
ERR2515066	ERR2515120	ERR2515171	ERR2515222	ERR2515273	ERR2515324	ERR2515375	ERR2515426

ERR2515427	ERR2515479	ERR2515536	ERR2515587	ERR2515638	ERR2515689	ERR2515740	ERR2515791
ERR2515428	ERR2515480	ERR2515537	ERR2515588	ERR2515639	ERR2515690	ERR2515741	ERR2515792
ERR2515429	ERR2515481	ERR2515538	ERR2515589	ERR2515640	ERR2515691	ERR2515742	ERR2515793
ERR2515430	ERR2515482	ERR2515539	ERR2515590	ERR2515641	ERR2515692	ERR2515743	ERR2515794
ERR2515431	ERR2515483	ERR2515540	ERR2515591	ERR2515642	ERR2515693	ERR2515744	ERR2515795
ERR2515432	ERR2515484	ERR2515541	ERR2515592	ERR2515643	ERR2515694	ERR2515745	ERR2515796
ERR2515433	ERR2515485	ERR2515542	ERR2515593	ERR2515644	ERR2515695	ERR2515746	ERR2515797
ERR2515434	ERR2515486	ERR2515543	ERR2515594	ERR2515645	ERR2515696	ERR2515747	ERR2515798
ERR2515435	ERR2515487	ERR2515544	ERR2515595	ERR2515646	ERR2515697	ERR2515748	ERR2515799
ERR2515436	ERR2515488	ERR2515545	ERR2515596	ERR2515647	ERR2515698	ERR2515749	ERR2515800
ERR2515437	ERR2515489	ERR2515546	ERR2515597	ERR2515648	ERR2515699	ERR2515750	ERR2515801
ERR2515438	ERR2515490	ERR2515547	ERR2515598	ERR2515649	ERR2515700	ERR2515751	ERR2515802
ERR2515439	ERR2515491	ERR2515548	ERR2515599	ERR2515650	ERR2515701	ERR2515752	ERR2515803
ERR2515440	ERR2515492	ERR2515549	ERR2515600	ERR2515651	ERR2515702	ERR2515753	ERR2515804
ERR2515441	ERR2515493	ERR2515550	ERR2515601	ERR2515652	ERR2515703	ERR2515754	ERR2515805
ERR2515442	ERR2515494	ERR2515551	ERR2515602	ERR2515653	ERR2515704	ERR2515755	ERR2515806
ERR2515443	ERR2515495	ERR2515552	ERR2515603	ERR2515654	ERR2515705	ERR2515756	ERR2515807
ERR2515444	ERR2515496	ERR2515553	ERR2515604	ERR2515655	ERR2515706	ERR2515757	ERR2515808
ERR2515445	ERR2515497	ERR2515554	ERR2515605	ERR2515656	ERR2515707	ERR2515758	ERR2515809
ERR2515446	ERR2515498	ERR2515555	ERR2515606	ERR2515657	ERR2515708	ERR2515759	ERR2515810
ERR2515447	ERR2515499	ERR2515556	ERR2515607	ERR2515658	ERR2515709	ERR2515760	ERR2515811
ERR2515448	ERR2515500	ERR2515557	ERR2515608	ERR2515659	ERR2515710	ERR2515761	ERR2515812
ERR2515449	ERR2515501	ERR2515558	ERR2515609	ERR2515660	ERR2515711	ERR2515762	ERR2515813
ERR2515450	ERR2515502	ERR2515559	ERR2515610	ERR2515661	ERR2515712	ERR2515763	ERR2515814
ERR2515451	ERR2515503	ERR2515560	ERR2515611	ERR2515662	ERR2515713	ERR2515764	ERR2515815
ERR2515452	ERR2515504	ERR2515561	ERR2515612	ERR2515663	ERR2515714	ERR2515765	ERR2515816
ERR2515453	ERR2515505	ERR2515562	ERR2515613	ERR2515664	ERR2515715	ERR2515766	ERR2515817
ERR2515454	ERR2515506	ERR2515563	ERR2515614	ERR2515665	ERR2515716	ERR2515767	ERR2515818
ERR2515456	ERR2515513	ERR2515564	ERR2515615	ERR2515666	ERR2515717	ERR2515768	ERR2515819
ERR2515457	ERR2515514	ERR2515565	ERR2515616	ERR2515667	ERR2515718	ERR2515769	ERR2515820
ERR2515458	ERR2515515	ERR2515566	ERR2515617	ERR2515668	ERR2515719	ERR2515770	ERR2515821
ERR2515459	ERR2515516	ERR2515567	ERR2515618	ERR2515669	ERR2515720	ERR2515771	ERR2515822
ERR2515460	ERR2515517	ERR2515568	ERR2515619	ERR2515670	ERR2515721	ERR2515772	ERR2515823
ERR2515461	ERR2515518	ERR2515569	ERR2515620	ERR2515671	ERR2515722	ERR2515773	ERR2515824
ERR2515462	ERR2515519	ERR2515570	ERR2515621	ERR2515672	ERR2515723	ERR2515774	ERR2515825
ERR2515463	ERR2515520	ERR2515571	ERR2515622	ERR2515673	ERR2515724	ERR2515775	ERR2515826
ERR2515464	ERR2515521	ERR2515572	ERR2515623	ERR2515674	ERR2515725	ERR2515776	ERR2515827
ERR2515465	ERR2515522	ERR2515573	ERR2515624	ERR2515675	ERR2515726	ERR2515777	ERR2515828
ERR2515466	ERR2515523	ERR2515574	ERR2515625	ERR2515676	ERR2515727	ERR2515778	ERR2515829
ERR2515467	ERR2515524	ERR2515575	ERR2515626	ERR2515677	ERR2515728	ERR2515779	ERR2515830
ERR2515468	ERR2515525	ERR2515576	ERR2515627	ERR2515678	ERR2515729	ERR2515780	ERR2515831
ERR2515469	ERR2515526	ERR2515577	ERR2515628	ERR2515679	ERR2515730	ERR2515781	ERR2515832
ERR2515470	ERR2515527	ERR2515578	ERR2515629	ERR2515680	ERR2515731	ERR2515782	ERR2515833
ERR2515471	ERR2515528	ERR2515579	ERR2515630	ERR2515681	ERR2515732	ERR2515783	ERR2515834
ERR2515472	ERR2515529	ERR2515580	ERR2515631	ERR2515682	ERR2515733	ERR2515784	ERR2515835
ERR2515473	ERR2515530	ERR2515581	ERR2515632	ERR2515683	ERR2515734	ERR2515785	ERR2515836
ERR2515474	ERR2515531	ERR2515582	ERR2515633	ERR2515684	ERR2515735	ERR2515786	ERR2515837
ERR2515475	ERR2515532	ERR2515583	ERR2515634	ERR2515685	ERR2515736	ERR2515787	ERR2515838
ERR2515476	ERR2515533	ERR2515584	ERR2515635	ERR2515686	ERR2515737	ERR2515788	ERR2515839
ERR2515477	ERR2515534	ERR2515585	ERR2515636	ERR2515687	ERR2515738	ERR2515789	ERR2515840
ERR2515478	ERR2515535	ERR2515586	ERR2515637	ERR2515688	ERR2515739	ERR2515790	ERR2515841

ERR2515842	ERR2515893	ERR2515944	ERR2515995	ERR2516046	ERR2516097	ERR2516149	ERR2516203
ERR2515843	ERR2515894	ERR2515945	ERR2515996	ERR2516047	ERR2516098	ERR2516150	ERR2516204
ERR2515844	ERR2515895	ERR2515946	ERR2515997	ERR2516048	ERR2516099	ERR2516151	ERR2516205
ERR2515845	ERR2515896	ERR2515947	ERR2515998	ERR2516049	ERR2516100	ERR2516152	ERR2516206
ERR2515846	ERR2515897	ERR2515948	ERR2515999	ERR2516050	ERR2516101	ERR2516153	ERR2516208
ERR2515847	ERR2515898	ERR2515949	ERR2516000	ERR2516051	ERR2516103	ERR2516154	ERR2516209
ERR2515848	ERR2515899	ERR2515950	ERR2516001	ERR2516052	ERR2516104	ERR2516155	ERR2516210
ERR2515849	ERR2515900	ERR2515951	ERR2516002	ERR2516053	ERR2516105	ERR2516156	ERR2516211
ERR2515850	ERR2515901	ERR2515952	ERR2516003	ERR2516054	ERR2516106	ERR2516157	ERR2516212
ERR2515851	ERR2515902	ERR2515953	ERR2516004	ERR2516055	ERR2516107	ERR2516158	ERR2516213
ERR2515852	ERR2515903	ERR2515954	ERR2516005	ERR2516056	ERR2516108	ERR2516159	ERR2516214
ERR2515853	ERR2515904	ERR2515955	ERR2516006	ERR2516057	ERR2516109	ERR2516160	ERR2516216
ERR2515854	ERR2515905	ERR2515956	ERR2516007	ERR2516058	ERR2516110	ERR2516161	ERR2516217
ERR2515855	ERR2515906	ERR2515957	ERR2516008	ERR2516059	ERR2516111	ERR2516162	ERR2516218
ERR2515856	ERR2515907	ERR2515958	ERR2516009	ERR2516060	ERR2516112	ERR2516163	ERR2516219
ERR2515857	ERR2515908	ERR2515959	ERR2516010	ERR2516061	ERR2516113	ERR2516164	ERR2516220
ERR2515858	ERR2515909	ERR2515960	ERR2516011	ERR2516062	ERR2516114	ERR2516165	ERR2516221
ERR2515859	ERR2515910	ERR2515961	ERR2516012	ERR2516063	ERR2516115	ERR2516166	ERR2516222
ERR2515860	ERR2515911	ERR2515962	ERR2516013	ERR2516064	ERR2516116	ERR2516167	ERR2516223
ERR2515861	ERR2515912	ERR2515963	ERR2516014	ERR2516065	ERR2516117	ERR2516168	ERR2516224
ERR2515862	ERR2515913	ERR2515964	ERR2516015	ERR2516066	ERR2516118	ERR2516169	ERR2516225
ERR2515863	ERR2515914	ERR2515965	ERR2516016	ERR2516067	ERR2516119	ERR2516171	ERR2516226
ERR2515864	ERR2515915	ERR2515966	ERR2516017	ERR2516068	ERR2516120	ERR2516172	ERR2516227
ERR2515865	ERR2515916	ERR2515967	ERR2516018	ERR2516069	ERR2516121	ERR2516173	ERR2516228
ERR2515866	ERR2515917	ERR2515968	ERR2516019	ERR2516070	ERR2516122	ERR2516174	ERR2516229
ERR2515867	ERR2515918	ERR2515969	ERR2516020	ERR2516071	ERR2516123	ERR2516175	ERR2516230
ERR2515868	ERR2515919	ERR2515970	ERR2516021	ERR2516072	ERR2516124	ERR2516176	ERR2516231
ERR2515869	ERR2515920	ERR2515971	ERR2516022	ERR2516073	ERR2516125	ERR2516177	ERR2516232
ERR2515870	ERR2515921	ERR2515972	ERR2516023	ERR2516074	ERR2516126	ERR2516178	ERR2516233
ERR2515871	ERR2515922	ERR2515973	ERR2516024	ERR2516075	ERR2516127	ERR2516179	ERR2516234
ERR2515872	ERR2515923	ERR2515974	ERR2516025	ERR2516076	ERR2516128	ERR2516180	ERR2516235
ERR2515873	ERR2515924	ERR2515975	ERR2516026	ERR2516077	ERR2516129	ERR2516181	ERR2516236
ERR2515874	ERR2515925	ERR2515976	ERR2516027	ERR2516078	ERR2516130	ERR2516182	ERR2516237
ERR2515875	ERR2515926	ERR2515977	ERR2516028	ERR2516079	ERR2516131	ERR2516183	ERR2516238
ERR2515876	ERR2515927	ERR2515978	ERR2516029	ERR2516080	ERR2516132	ERR2516184	ERR2516239
ERR2515877	ERR2515928	ERR2515979	ERR2516030	ERR2516081	ERR2516133	ERR2516185	ERR2516240
ERR2515878	ERR2515929	ERR2515980	ERR2516031	ERR2516082	ERR2516134	ERR2516186	ERR2516241
ERR2515879	ERR2515930	ERR2515981	ERR2516032	ERR2516083	ERR2516135	ERR2516187	ERR2516242
ERR2515880	ERR2515931	ERR2515982	ERR2516033	ERR2516084	ERR2516136	ERR2516188	ERR2516243
ERR2515881	ERR2515932	ERR2515983	ERR2516034	ERR2516085	ERR2516137	ERR2516190	ERR2516244
ERR2515882	ERR2515933	ERR2515984	ERR2516035	ERR2516086	ERR2516138	ERR2516192	ERR2516245
ERR2515883	ERR2515934	ERR2515985	ERR2516036	ERR2516087	ERR2516139	ERR2516193	ERR2516246
ERR2515884	ERR2515935	ERR2515986	ERR2516037	ERR2516088	ERR2516140	ERR2516194	ERR2516247
ERR2515885	ERR2515936	ERR2515987	ERR2516038	ERR2516089	ERR2516141	ERR2516195	ERR2516248
ERR2515886	ERR2515937	ERR2515988	ERR2516039	ERR2516090	ERR2516142	ERR2516196	ERR2516249
ERR2515887	ERR2515938	ERR2515989	ERR2516040	ERR2516091	ERR2516143	ERR2516197	ERR2516250
ERR2515888	ERR2515939	ERR2515990	ERR2516041	ERR2516092	ERR2516144	ERR2516198	ERR2516251
ERR2515889	ERR2515940	ERR2515991	ERR2516042	ERR2516093	ERR2516145	ERR2516199	ERR2516253
ERR2515890	ERR2515941	ERR2515992	ERR2516043	ERR2516094	ERR2516146	ERR2516200	ERR2516254
ERR2515891	ERR2515942	ERR2515993	ERR2516044	ERR2516095	ERR2516147	ERR2516201	ERR2516255
ERR2515892	ERR2515943	ERR2515994	ERR2516045	ERR2516096	ERR2516148	ERR2516202	ERR2516256

ERR2516257	ERR2516310	ERR2516363	ERR2516416	ERR2516468	ERR2516519	ERR2516571	ERR2516622
ERR2516258	ERR2516312	ERR2516364	ERR2516417	ERR2516469	ERR2516520	ERR2516572	ERR2516623
ERR2516259	ERR2516313	ERR2516365	ERR2516418	ERR2516470	ERR2516521	ERR2516573	ERR2516624
ERR2516260	ERR2516314	ERR2516366	ERR2516419	ERR2516471	ERR2516522	ERR2516574	ERR2516625
ERR2516261	ERR2516315	ERR2516367	ERR2516420	ERR2516472	ERR2516523	ERR2516575	ERR2516626
ERR2516263	ERR2516316	ERR2516368	ERR2516421	ERR2516473	ERR2516524	ERR2516576	ERR2516627
ERR2516264	ERR2516317	ERR2516369	ERR2516422	ERR2516474	ERR2516525	ERR2516577	ERR2516628
ERR2516265	ERR2516318	ERR2516370	ERR2516423	ERR2516475	ERR2516526	ERR2516578	ERR2516629
ERR2516266	ERR2516319	ERR2516371	ERR2516424	ERR2516476	ERR2516527	ERR2516579	ERR2516630
ERR2516267	ERR2516320	ERR2516372	ERR2516425	ERR2516477	ERR2516528	ERR2516580	ERR2516631
ERR2516268	ERR2516321	ERR2516373	ERR2516426	ERR2516478	ERR2516529	ERR2516581	ERR2516632
ERR2516269	ERR2516322	ERR2516374	ERR2516427	ERR2516479	ERR2516530	ERR2516582	ERR2516633
ERR2516270	ERR2516323	ERR2516375	ERR2516428	ERR2516480	ERR2516531	ERR2516583	ERR2516634
ERR2516271	ERR2516324	ERR2516377	ERR2516429	ERR2516481	ERR2516532	ERR2516584	ERR2516635
ERR2516272	ERR2516325	ERR2516378	ERR2516430	ERR2516482	ERR2516533	ERR2516585	ERR2516636
ERR2516273	ERR2516326	ERR2516379	ERR2516431	ERR2516483	ERR2516534	ERR2516586	ERR2516637
ERR2516274	ERR2516327	ERR2516380	ERR2516432	ERR2516484	ERR2516535	ERR2516587	ERR2516638
ERR2516275	ERR2516328	ERR2516381	ERR2516433	ERR2516485	ERR2516536	ERR2516588	ERR2516639
ERR2516276	ERR2516329	ERR2516382	ERR2516434	ERR2516486	ERR2516537	ERR2516589	ERR2516640
ERR2516277	ERR2516330	ERR2516383	ERR2516435	ERR2516487	ERR2516538	ERR2516590	ERR2516641
ERR2516278	ERR2516331	ERR2516384	ERR2516436	ERR2516488	ERR2516539	ERR2516591	ERR2516642
ERR2516279	ERR2516332	ERR2516385	ERR2516437	ERR2516489	ERR2516540	ERR2516592	ERR2516643
ERR2516280	ERR2516333	ERR2516386	ERR2516438	ERR2516490	ERR2516541	ERR2516593	ERR2516644
ERR2516281	ERR2516334	ERR2516387	ERR2516439	ERR2516491	ERR2516542	ERR2516594	ERR2516645
ERR2516282	ERR2516336	ERR2516388	ERR2516440	ERR2516492	ERR2516543	ERR2516595	ERR2516646
ERR2516283	ERR2516337	ERR2516389	ERR2516441	ERR2516493	ERR2516544	ERR2516596	ERR2516647
ERR2516284	ERR2516338	ERR2516390	ERR2516442	ERR2516494	ERR2516546	ERR2516597	ERR2516648
ERR2516285	ERR2516339	ERR2516391	ERR2516443	ERR2516495	ERR2516547	ERR2516598	ERR2516649
ERR2516286	ERR2516340	ERR2516392	ERR2516444	ERR2516496	ERR2516548	ERR2516599	ERR2516650
ERR2516287	ERR2516341	ERR2516393	ERR2516445	ERR2516497	ERR2516549	ERR2516600	ERR2516651
ERR2516288	ERR2516342	ERR2516394	ERR2516446	ERR2516498	ERR2516550	ERR2516601	ERR2516652
ERR2516289	ERR2516343	ERR2516395	ERR2516447	ERR2516499	ERR2516551	ERR2516602	ERR2516653
ERR2516290	ERR2516344	ERR2516396	ERR2516448	ERR2516500	ERR2516552	ERR2516603	ERR2516654
ERR2516291	ERR2516345	ERR2516397	ERR2516449	ERR2516501	ERR2516553	ERR2516604	ERR2516655
ERR2516292	ERR2516346	ERR2516398	ERR2516450	ERR2516502	ERR2516554	ERR2516605	ERR2516656
ERR2516293	ERR2516347	ERR2516399	ERR2516451	ERR2516503	ERR2516555	ERR2516606	ERR2516657
ERR2516294	ERR2516348	ERR2516400	ERR2516452	ERR2516504	ERR2516556	ERR2516607	ERR2516658
ERR2516295	ERR2516349	ERR2516402	ERR2516454	ERR2516505	ERR2516557	ERR2516608	ERR2516659
ERR2516296	ERR2516350	ERR2516403	ERR2516455	ERR2516506	ERR2516558	ERR2516609	ERR2516660
ERR2516297	ERR2516351	ERR2516404	ERR2516456	ERR2516507	ERR2516559	ERR2516610	ERR2516661
ERR2516298	ERR2516352	ERR2516405	ERR2516457	ERR2516508	ERR2516560	ERR2516611	ERR2516662
ERR2516299	ERR2516353	ERR2516406	ERR2516458	ERR2516509	ERR2516561	ERR2516612	ERR2516663
ERR2516300	ERR2516354	ERR2516407	ERR2516459	ERR2516510	ERR2516562	ERR2516613	ERR2516664
ERR2516301	ERR2516355	ERR2516408	ERR2516460	ERR2516511	ERR2516563	ERR2516614	ERR2516665
ERR2516302	ERR2516356	ERR2516409	ERR2516461	ERR2516512	ERR2516564	ERR2516615	ERR2516666
ERR2516303	ERR2516357	ERR2516410	ERR2516462	ERR2516513	ERR2516565	ERR2516616	ERR2516667
ERR2516304	ERR2516358	ERR2516411	ERR2516463	ERR2516514	ERR2516566	ERR2516617	ERR2516668
ERR2516305	ERR2516359	ERR2516412	ERR2516464	ERR2516515	ERR2516567	ERR2516618	ERR2516669
ERR2516306	ERR2516360	ERR2516413	ERR2516465	ERR2516516	ERR2516568	ERR2516619	ERR2516670
ERR2516308	ERR2516361	ERR2516414	ERR2516466	ERR2516517	ERR2516569	ERR2516620	ERR2516671
ERR2516309	ERR2516362	ERR2516415	ERR2516467	ERR2516518	ERR2516570	ERR2516621	ERR2516672

ERR2516673	ERR2516724	ERR2516777	ERR2516828	ERR2516879	ERR2516930	ERR2516981	ERR2517032
ERR2516674	ERR2516725	ERR2516778	ERR2516829	ERR2516880	ERR2516931	ERR2516982	ERR2517033
ERR2516675	ERR2516726	ERR2516779	ERR2516830	ERR2516881	ERR2516932	ERR2516983	ERR2517034
ERR2516676	ERR2516727	ERR2516780	ERR2516831	ERR2516882	ERR2516933	ERR2516984	ERR2517035
ERR2516677	ERR2516728	ERR2516781	ERR2516832	ERR2516883	ERR2516934	ERR2516985	ERR2517036
ERR2516678	ERR2516729	ERR2516782	ERR2516833	ERR2516884	ERR2516935	ERR2516986	ERR2517037
ERR2516679	ERR2516730	ERR2516783	ERR2516834	ERR2516885	ERR2516936	ERR2516987	ERR2517038
ERR2516680	ERR2516731	ERR2516784	ERR2516835	ERR2516886	ERR2516937	ERR2516988	ERR2517039
ERR2516681	ERR2516732	ERR2516785	ERR2516836	ERR2516887	ERR2516938	ERR2516989	ERR2517040
ERR2516682	ERR2516733	ERR2516786	ERR2516837	ERR2516888	ERR2516939	ERR2516990	ERR2517041
ERR2516683	ERR2516734	ERR2516787	ERR2516838	ERR2516889	ERR2516940	ERR2516991	ERR2517042
ERR2516684	ERR2516735	ERR2516788	ERR2516839	ERR2516890	ERR2516941	ERR2516992	ERR2517043
ERR2516685	ERR2516736	ERR2516789	ERR2516840	ERR2516891	ERR2516942	ERR2516993	ERR2517044
ERR2516686	ERR2516737	ERR2516790	ERR2516841	ERR2516892	ERR2516943	ERR2516994	ERR2517045
ERR2516687	ERR2516738	ERR2516791	ERR2516842	ERR2516893	ERR2516944	ERR2516995	ERR2517046
ERR2516688	ERR2516740	ERR2516792	ERR2516843	ERR2516894	ERR2516945	ERR2516996	ERR2517047
ERR2516689	ERR2516741	ERR2516793	ERR2516844	ERR2516895	ERR2516946	ERR2516997	ERR2517048
ERR2516690	ERR2516742	ERR2516794	ERR2516845	ERR2516896	ERR2516947	ERR2516998	ERR2517049
ERR2516691	ERR2516744	ERR2516795	ERR2516846	ERR2516897	ERR2516948	ERR2516999	ERR2517050
ERR2516692	ERR2516745	ERR2516796	ERR2516847	ERR2516898	ERR2516949	ERR2517000	ERR2517051
ERR2516693	ERR2516746	ERR2516797	ERR2516848	ERR2516899	ERR2516950	ERR2517001	ERR2517052
ERR2516694	ERR2516747	ERR2516798	ERR2516849	ERR2516900	ERR2516951	ERR2517002	ERR2517053
ERR2516695	ERR2516748	ERR2516799	ERR2516850	ERR2516901	ERR2516952	ERR2517003	ERR2517054
ERR2516696	ERR2516749	ERR2516800	ERR2516851	ERR2516902	ERR2516953	ERR2517004	ERR2517055
ERR2516697	ERR2516750	ERR2516801	ERR2516852	ERR2516903	ERR2516954	ERR2517005	ERR2517056
ERR2516698	ERR2516751	ERR2516802	ERR2516853	ERR2516904	ERR2516955	ERR2517006	ERR2517057
ERR2516699	ERR2516752	ERR2516803	ERR2516854	ERR2516905	ERR2516956	ERR2517007	ERR2517058
ERR2516700	ERR2516753	ERR2516804	ERR2516855	ERR2516906	ERR2516957	ERR2517008	ERR2517059
ERR2516701	ERR2516754	ERR2516805	ERR2516856	ERR2516907	ERR2516958	ERR2517009	ERR2517060
ERR2516702	ERR2516755	ERR2516806	ERR2516857	ERR2516908	ERR2516959	ERR2517010	ERR2517061
ERR2516703	ERR2516756	ERR2516807	ERR2516858	ERR2516909	ERR2516960	ERR2517011	ERR2517062
ERR2516704	ERR2516757	ERR2516808	ERR2516859	ERR2516910	ERR2516961	ERR2517012	ERR2517063
ERR2516705	ERR2516758	ERR2516809	ERR2516860	ERR2516911	ERR2516962	ERR2517013	ERR2517064
ERR2516706	ERR2516759	ERR2516810	ERR2516861	ERR2516912	ERR2516963	ERR2517014	ERR2517065
ERR2516707	ERR2516760	ERR2516811	ERR2516862	ERR2516913	ERR2516964	ERR2517015	ERR2517066
ERR2516708	ERR2516761	ERR2516812	ERR2516863	ERR2516914	ERR2516965	ERR2517016	ERR2517067
ERR2516709	ERR2516762	ERR2516813	ERR2516864	ERR2516915	ERR2516966	ERR2517017	ERR2517068
ERR2516710	ERR2516763	ERR2516814	ERR2516865	ERR2516916	ERR2516967	ERR2517018	ERR2517069
ERR2516711	ERR2516764	ERR2516815	ERR2516866	ERR2516917	ERR2516968	ERR2517019	ERR2517070
ERR2516712	ERR2516765	ERR2516816	ERR2516867	ERR2516918	ERR2516969	ERR2517020	ERR2517071
ERR2516713	ERR2516766	ERR2516817	ERR2516868	ERR2516919	ERR2516970	ERR2517021	ERR2517072
ERR2516714	ERR2516767	ERR2516818	ERR2516869	ERR2516920	ERR2516971	ERR2517022	ERR2517073
ERR2516715	ERR2516768	ERR2516819	ERR2516870	ERR2516921	ERR2516972	ERR2517023	ERR2517074
ERR2516716	ERR2516769	ERR2516820	ERR2516871	ERR2516922	ERR2516973	ERR2517024	ERR2517075
ERR2516717	ERR2516770	ERR2516821	ERR2516872	ERR2516923	ERR2516974	ERR2517025	ERR2517076
ERR2516718	ERR2516771	ERR2516822	ERR2516873	ERR2516924	ERR2516975	ERR2517026	ERR2517077
ERR2516719	ERR2516772	ERR2516823	ERR2516874	ERR2516925	ERR2516976	ERR2517027	ERR2517078
ERR2516720	ERR2516773	ERR2516824	ERR2516875	ERR2516926	ERR2516977	ERR2517028	ERR2517079
ERR2516721	ERR2516774	ERR2516825	ERR2516876	ERR2516927	ERR2516978	ERR2517029	ERR2517080
ERR2516722	ERR2516775	ERR2516826	ERR2516877	ERR2516928	ERR2516979	ERR2517030	ERR2517081
ERR2516723	ERR2516776	ERR2516827	ERR2516878	ERR2516929	ERR2516980	ERR2517031	ERR2517082

ERR2517083	ERR2517136	ERR2517190	ERR2517242	ERR2517293	ERR2517344	ERR2517399	ERR2517453
ERR2517084	ERR2517137	ERR2517191	ERR2517243	ERR2517294	ERR2517346	ERR2517400	ERR2517454
ERR2517085	ERR2517138	ERR2517192	ERR2517244	ERR2517295	ERR2517347	ERR2517401	ERR2517455
ERR2517086	ERR2517139	ERR2517193	ERR2517245	ERR2517296	ERR2517348	ERR2517402	ERR2517456
ERR2517087	ERR2517140	ERR2517194	ERR2517246	ERR2517297	ERR2517350	ERR2517403	ERR2517457
ERR2517088	ERR2517141	ERR2517195	ERR2517247	ERR2517298	ERR2517351	ERR2517404	ERR2517458
ERR2517089	ERR2517142	ERR2517196	ERR2517248	ERR2517299	ERR2517352	ERR2517405	ERR2517459
ERR2517090	ERR2517143	ERR2517197	ERR2517249	ERR2517300	ERR2517353	ERR2517406	ERR2517460
ERR2517091	ERR2517144	ERR2517198	ERR2517250	ERR2517301	ERR2517354	ERR2517407	ERR2517461
ERR2517092	ERR2517145	ERR2517199	ERR2517251	ERR2517302	ERR2517355	ERR2517409	ERR2517462
ERR2517093	ERR2517146	ERR2517200	ERR2517252	ERR2517303	ERR2517356	ERR2517410	ERR2517463
ERR2517094	ERR2517147	ERR2517201	ERR2517253	ERR2517304	ERR2517358	ERR2517411	ERR2517464
ERR2517095	ERR2517148	ERR2517202	ERR2517254	ERR2517305	ERR2517359	ERR2517412	ERR2517465
ERR2517096	ERR2517149	ERR2517203	ERR2517255	ERR2517306	ERR2517360	ERR2517413	ERR2517466
ERR2517097	ERR2517150	ERR2517204	ERR2517256	ERR2517307	ERR2517361	ERR2517414	ERR2517467
ERR2517098	ERR2517152	ERR2517205	ERR2517257	ERR2517308	ERR2517362	ERR2517415	ERR2517468
ERR2517099	ERR2517153	ERR2517206	ERR2517258	ERR2517309	ERR2517363	ERR2517416	ERR2517469
ERR2517100	ERR2517154	ERR2517207	ERR2517259	ERR2517310	ERR2517364	ERR2517417	ERR2517470
ERR2517101	ERR2517155	ERR2517208	ERR2517260	ERR2517311	ERR2517365	ERR2517418	ERR2517471
ERR2517103	ERR2517156	ERR2517209	ERR2517261	ERR2517312	ERR2517366	ERR2517419	ERR2517472
ERR2517104	ERR2517157	ERR2517210	ERR2517262	ERR2517313	ERR2517367	ERR2517420	ERR2517473
ERR2517105	ERR2517158	ERR2517211	ERR2517263	ERR2517314	ERR2517369	ERR2517421	ERR2517474
ERR2517106	ERR2517159	ERR2517212	ERR2517264	ERR2517315	ERR2517370	ERR2517422	ERR2517475
ERR2517107	ERR2517160	ERR2517213	ERR2517265	ERR2517316	ERR2517371	ERR2517423	ERR2517476
ERR2517108	ERR2517161	ERR2517214	ERR2517266	ERR2517317	ERR2517372	ERR2517424	ERR2517477
ERR2517109	ERR2517162	ERR2517215	ERR2517267	ERR2517318	ERR2517373	ERR2517426	ERR2517478
ERR2517110	ERR2517163	ERR2517216	ERR2517268	ERR2517319	ERR2517374	ERR2517427	ERR2517479
ERR2517111	ERR2517164	ERR2517217	ERR2517269	ERR2517320	ERR2517375	ERR2517428	ERR2517480
ERR2517112	ERR2517165	ERR2517218	ERR2517270	ERR2517321	ERR2517376	ERR2517429	ERR2517481
ERR2517113	ERR2517167	ERR2517219	ERR2517271	ERR2517322	ERR2517377	ERR2517430	ERR2517482
ERR2517114	ERR2517168	ERR2517220	ERR2517272	ERR2517323	ERR2517378	ERR2517431	ERR2517483
ERR2517115	ERR2517169	ERR2517221	ERR2517273	ERR2517324	ERR2517379	ERR2517432	ERR2517484
ERR2517116	ERR2517170	ERR2517222	ERR2517274	ERR2517325	ERR2517380	ERR2517433	ERR2517485
ERR2517117	ERR2517171	ERR2517223	ERR2517275	ERR2517326	ERR2517381	ERR2517434	ERR2517486
ERR2517118	ERR2517172	ERR2517224	ERR2517276	ERR2517327	ERR2517382	ERR2517435	ERR2517487
ERR2517119	ERR2517173	ERR2517225	ERR2517277	ERR2517328	ERR2517383	ERR2517436	ERR2517488
ERR2517120	ERR2517174	ERR2517226	ERR2517278	ERR2517329	ERR2517384	ERR2517437	ERR2517489
ERR2517121	ERR2517175	ERR2517228	ERR2517279	ERR2517330	ERR2517385	ERR2517438	ERR2517490
ERR2517122	ERR2517176	ERR2517229	ERR2517280	ERR2517331	ERR2517386	ERR2517439	ERR2517491
ERR2517123	ERR2517177	ERR2517230	ERR2517281	ERR2517332	ERR2517387	ERR2517440	ERR2517492
ERR2517124	ERR2517178	ERR2517231	ERR2517282	ERR2517333	ERR2517388	ERR2517441	ERR2517493
ERR2517125	ERR2517180	ERR2517232	ERR2517283	ERR2517334	ERR2517389	ERR2517442	ERR2517494
ERR2517126	ERR2517181	ERR2517233	ERR2517284	ERR2517335	ERR2517390	ERR2517444	ERR2517496
ERR2517127	ERR2517182	ERR2517234	ERR2517285	ERR2517336	ERR2517391	ERR2517445	ERR2517497
ERR2517128	ERR2517183	ERR2517235	ERR2517286	ERR2517337	ERR2517392	ERR2517446	ERR2517498
ERR2517130	ERR2517184	ERR2517236	ERR2517287	ERR2517338	ERR2517393	ERR2517447	ERR2517499
ERR2517131	ERR2517185	ERR2517237	ERR2517288	ERR2517339	ERR2517394	ERR2517448	ERR2517500
ERR2517132	ERR2517186	ERR2517238	ERR2517289	ERR2517340	ERR2517395	ERR2517449	ERR2517501
ERR2517133	ERR2517187	ERR2517239	ERR2517290	ERR2517341	ERR2517396	ERR2517450	ERR2517502
ERR2517134	ERR2517188	ERR2517240	ERR2517291	ERR2517342	ERR2517397	ERR2517451	ERR2517503
ERR2517135	ERR2517189	ERR2517241	ERR2517292	ERR2517343	ERR2517398	ERR2517452	ERR2517504

ERR2517505	ERR2517558	ERR2517610	SRR671729	SRR671798	SRR671856	SRR3675573	SRR5709794
ERR2517506	ERR2517559	ERR2517611	SRR671730	SRR671799	SRR671857	SRR3675590	SRR5709795
ERR2517507	ERR2517560	ERR2517612	SRR671732	SRR671800	SRR671858	SRR5709738	SRR5709796
ERR2517508	ERR2517561	ERR2517613	SRR671733	SRR671801	SRR671859	SRR5709739	SRR5709797
ERR2517509	ERR2517562	ERR2517614	SRR671734	SRR671802	SRR671860	SRR5709740	SRR5709798
ERR2517510	ERR2517563	ERR2517615	SRR671735	SRR671804	SRR671861	SRR5709741	SRR5709799
ERR2517511	ERR2517564	ERR2517617	SRR671736	SRR671805	SRR671862	SRR5709742	SRR5709801
ERR2517512	ERR2517565	ERR2517618	SRR671737	SRR671806	SRR671863	SRR5709744	SRR5709802
ERR2517513	ERR2517566	ERR2517619	SRR671738	SRR671807	SRR671864	SRR5709746	SRR5709803
ERR2517514	ERR2517567	ERR2517620	SRR671739	SRR671808	SRR671865	SRR5709747	SRR5709804
ERR2517515	ERR2517568	ERR2517621	SRR671741	SRR671809	SRR671866	SRR5709748	SRR5709805
ERR2517516	ERR2517569	ERR2517622	SRR671742	SRR671810	SRR671867	SRR5709749	SRR5709806
ERR2517517	ERR2517570	ERR2517623	SRR671743	SRR671811	SRR671868	SRR5709750	SRR5709807
ERR2517519	ERR2517571	ERR2517624	SRR671745	SRR671812	SRR671869	SRR5709751	SRR5709808
ERR2517520	ERR2517572	ERR2517625	SRR671746	SRR671813	SRR671870	SRR5709752	SRR5709809
ERR2517521	ERR2517573	ERR2517626	SRR671747	SRR671814	SRR671871	SRR5709754	SRR5709810
ERR2517522	ERR2517574	ERR2517627	SRR671748	SRR671815	SRR671874	SRR5709755	SRR5709811
ERR2517524	ERR2517575	ERR2517628	SRR671749	SRR671816	SRR671876	SRR5709756	SRR5709812
ERR2517525	ERR2517576	ERR2517629	SRR671750	SRR671818	SRR671877	SRR5709757	SRR5709813
ERR2517526	ERR2517577	ERR2517630	SRR671751	SRR671819	SRR671878	SRR5709758	SRR5709814
ERR2517527	ERR2517578	ERR2517631	SRR671752	SRR671820	SRR2328057	SRR5709759	SRR5709815
ERR2517528	ERR2517579	ERR2517632	SRR671753	SRR671822	SRR2333215	SRR5709760	SRR5709816
ERR2517529	ERR2517580	ERR2517633	SRR671754	SRR671823	SRR3675225	SRR5709761	SRR5709817
ERR2517530	ERR2517581	ERR2517634	SRR671755	SRR671824	SRR3675238	SRR5709762	SRR5709818
ERR2517531	ERR2517582	ERR2517635	SRR671756	SRR671825	SRR3675239	SRR5709763	SRR5709819
ERR2517532	ERR2517583	ERR2517636	SRR671757	SRR671826	SRR3675242	SRR5709764	SRR5709820
ERR2517533	ERR2517584	ERR2517637	SRR671759	SRR671828	SRR3675244	SRR5709765	SRR5709823
ERR2517534	ERR2517585	ERR2517638	SRR671762	SRR671829	SRR3675250	SRR5709766	SRR5709824
ERR2517535	ERR2517586	ERR2517639	SRR671764	SRR671831	SRR3675252	SRR5709767	SRR5709825
ERR2517536	ERR2517587	ERR2517640	SRR671765	SRR671833	SRR3675255	SRR5709768	SRR5709826
ERR2517537	ERR2517588	ERR2517641	SRR671766	SRR671834	SRR3675261	SRR5709769	SRR5709832
ERR2517538	ERR2517589	ERR2517642	SRR671768	SRR671835	SRR3675262	SRR5709770	SRR5709837
ERR2517539	ERR2517590	ERR2517643	SRR671769	SRR671836	SRR3675273	SRR5709772	SRR5709839
ERR2517540	ERR2517591	ERR2517644	SRR671771	SRR671837	SRR3675274	SRR5709773	SRR5709840
ERR2517541	ERR2517592	ERR2517645	SRR671773	SRR671838	SRR3675277	SRR5709774	SRR5709841
ERR2517542	ERR2517594	ERR2517646	SRR671775	SRR671840	SRR3675286	SRR5709775	SRR5709842
ERR2517543	ERR2517595	ERR2517647	SRR671777	SRR671841	SRR3675289	SRR5709776	SRR5709843
ERR2517544	ERR2517596	ERR2517648	SRR671778	SRR671842	SRR3675314	SRR5709777	SRR5709844
ERR2517545	ERR2517597	ERR2517649	SRR671780	SRR671843	SRR3675336	SRR5709778	SRR5709845
ERR2517546	ERR2517598	ERR2517650	SRR671784	SRR671844	SRR3675391	SRR5709779	SRR5709846
ERR2517547	ERR2517599	ERR2517651	SRR671785	SRR671845	SRR3675439	SRR5709780	SRR5709847
ERR2517548	ERR2517600	ERR2517652	SRR671786	SRR671846	SRR3675458	SRR5709781	SRR5709848
ERR2517549	ERR2517601	ERR2517653	SRR671787	SRR671847	SRR3675466	SRR5709783	SRR5709849
ERR2517550	ERR2517602	SRR671720	SRR671788	SRR671848	SRR3675469	SRR5709784	SRR5709852
ERR2517551	ERR2517603	SRR671722	SRR671789	SRR671849	SRR3675489	SRR5709785	SRR5709853
ERR2517552	ERR2517604	SRR671723	SRR671790	SRR671850	SRR3675523	SRR5709786	SRR5709854
ERR2517553	ERR2517605	SRR671724	SRR671792	SRR671851	SRR3675530	SRR5709787	SRR5709855
ERR2517554	ERR2517606	SRR671725	SRR671793	SRR671852	SRR3675531	SRR5709788	SRR5709858
ERR2517555	ERR2517607	SRR671726	SRR671795	SRR671853	SRR3675532	SRR5709789	SRR5709859
ERR2517556	ERR2517608	SRR671727	SRR671796	SRR671854	SRR3675533	SRR5709792	SRR5709860
ERR2517557	ERR2517609	SRR671728	SRR671797	SRR671855	SRR3675538	SRR5709793	SRR5709861

SRR5709862	SRR5709919	SRR5709980	SRR5817473	TB0037	TB0107	TB0170	TB0234
SRR5709863	SRR5709920	SRR5709981	SRR5817475	TB0038	TB0108	TB0171	TB0235
SRR5709864	SRR5709922	SRR5709982	SRR5837706	TB0039	TB0109	TB0172	TB0236
SRR5709865	SRR5709923	SRR5709983	SRR5837707	TB0040	TB0110	TB0173	TB0237
SRR5709866	SRR5709924	SRR5709984	SRR5837708	TB0041	TB0111	TB0175	TB0238
SRR5709867	SRR5709925	SRR5709985	SRR5837709	TB0042	TB0113	TB0176	TB0239
SRR5709870	SRR5709926	SRR5709986	SRR5837710	TB0043	TB0114	TB0177	TB0240
SRR5709872	SRR5709927	SRR5709989	SRR5837711	TB0044	TB0116	TB0178	TB0241
SRR5709873	SRR5709929	SRR5709990	SRR5837712	TB0045	TB0117	TB0179	TB0242
SRR5709874	SRR5709930	SRR5709991	SRR6367398	TB0046	TB0118	TB0181	TB0243
SRR5709876	SRR5709932	SRR5709992	SRR6367399	TB0047	TB0119	TB0182	TB0244
SRR5709877	SRR5709933	SRR5709993	SRR6367400	TB0048	TB0120	TB0183	TB0245
SRR5709878	SRR5709934	SRR5709994	SRR6367401	TB0049	TB0121	TB0184	TB0246
SRR5709879	SRR5709935	SRR5709996	SRR6369875	TB0050	TB0122	TB0185	TB0250
SRR5709880	SRR5709936	SRR5709997	SRR6369876	TB0051	TB0123	TB0186	TB0251
SRR5709881	SRR5709938	SRR5709998	SRR6369877	TB0052	TB0124	TB0188	TB0253
SRR5709882	SRR5709940	SRR5709999	SRR6369878	TB0053	TB0125	TB0189	TB0254
SRR5709883	SRR5709941	SRR5710001	TB0001	TB0054	TB0126	TB0191	TB0255
SRR5709884	SRR5709942	SRR5710002	TB0002	TB0055	TB0127	TB0194	TB0256
SRR5709885	SRR5709943	SRR5710003	TB0003	TB0056	TB0128	TB0195	TB0257
SRR5709886	SRR5709944	SRR5710005	TB0004	TB0057	TB0129	TB0196	TB0258
SRR5709887	SRR5709945	SRR5710006	TB0005	TB0058	TB0131	TB0197	TB0259
SRR5709888	SRR5709946	SRR5710007	TB0006	TB0060	TB0132	TB0198	TB0261
SRR5709889	SRR5709947	SRR5710008	TB0008	TB0061	TB0133	TB0199	TB0262
SRR5709890	SRR5709949	SRR5710009	TB0009	TB0062	TB0134	TB0200	TB0263
SRR5709891	SRR5709950	SRR5710010	TB0010	TB0063	TB0135	TB0201	TB0265
SRR5709892	SRR5709951	SRR5710011	TB0011	TB0064	TB0138	TB0202	TB0266
SRR5709893	SRR5709952	SRR5710012	TB0012	TB0065	TB0140	TB0203	TB0267
SRR5709894	SRR5709953	SRR5710013	TB0013	TB0066	TB0141	TB0204	TB0268
SRR5709895	SRR5709954	SRR5710014	TB0014	TB0067	TB0142	TB0205	TB0269
SRR5709896	SRR5709956	SRR5710015	TB0015	TB0068	TB0143	TB0206	TB0270
SRR5709897	SRR5709957	SRR5710016	TB0016	TB0069	TB0146	TB0207	TB0271
SRR5709898	SRR5709958	SRR5710017	TB0017	TB0070	TB0148	TB0208	TB0272
SRR5709899	SRR5709959	SRR5710018	TB0018	TB0071	TB0149	TB0209	TB0273
SRR5709900	SRR5709960	SRR5710019	TB0019	TB0072	TB0150	TB0211	TB0274
SRR5709901	SRR5709961	SRR5710020	TB0020	TB0073	TB0151	TB0214	TB0276
SRR5709902	SRR5709962	SRR5710022	TB0021	TB0074	TB0152	TB0215	TB0277
SRR5709903	SRR5709963	SRR5710023	TB0022	TB0075	TB0154	TB0216	TB0278
SRR5709904	SRR5709964	SRR5710024	TB0023	TB0088	TB0155	TB0217	TB0312
SRR5709905	SRR5709965	SRR5710025	TB0024	TB0089	TB0156	TB0219	TB0313
SRR5709906	SRR5709968	SRR5710026	TB0025	TB0090	TB0158	TB0220	TB0314
SRR5709907	SRR5709969	SRR5710027	TB0027	TB0091	TB0159	TB0222	TB0315
SRR5709909	SRR5709970	SRR5710028	TB0028	TB0092	TB0160	TB0224	TB0316
SRR5709910	SRR5709971	SRR5817463	TB0029	TB0095	TB0161	TB0226	TB0317
SRR5709911	SRR5709972	SRR5817464	TB0030	TB0099	TB0162	TB0227	TB0318
SRR5709912	SRR5709973	SRR5817466	TB0031	TB0100	TB0163	TB0228	TB0319
SRR5709913	SRR5709974	SRR5817467	TB0032	TB0102	TB0164	TB0229	TB0320
SRR5709915	SRR5709976	SRR5817469	TB0033	TB0103	TB0165	TB0230	TB0321
SRR5709916	SRR5709977	SRR5817470	TB0034	TB0104	TB0166	TB0231	TB0322
SRR5709917	SRR5709978	SRR5817471	TB0035	TB0105	TB0167	TB0232	TB0323
SRR5709918	SRR5709979	SRR5817472	TB0036	TB0106	TB0169	TB0233	TB0324

TB0325	TB0404	TB0490	TB0563	TB0625	TB0692	TB0757	TB0822
TB0327	TB0406	TB0491	TB0564	TB0628	TB0693	TB0758	TB0823
TB0328	TB0407	TB0493	TB0565	TB0629	TB0694	TB0761	TB0824
TB0330	TB0408	TB0494	TB0566	TB0632	TB0695	TB0763	TB0825
TB0331	TB0409	TB0497	TB0567	TB0633	TB0696	TB0765	TB0826
TB0334	TB0410	TB0501	TB0568	TB0634	TB0697	TB0766	TB0827
TB0335	TB0411	TB0502	TB0569	TB0636	TB0698	TB0767	TB0829
TB0337	TB0413	TB0504	TB0570	TB0637	TB0699	TB0768	TB0830
TB0338	TB0414	TB0505	TB0573	TB0638	TB0700	TB0770	TB0831
TB0340	TB0415	TB0506	TB0574	TB0639	TB0701	TB0771	TB0835
TB0341	TB0416	TB0507	TB0575	TB0640	TB0702	TB0772	TB0836
TB0342	TB0418	TB0509	TB0577	TB0643	TB0703	TB0773	TB0837
TB0344	TB0419	TB0510	TB0578	TB0644	TB0704	TB0774	TB0840
TB0345	TB0420	TB0513	TB0579	TB0646	TB0705	TB0775	TB0841
TB0346	TB0421	TB0514	TB0580	TB0647	TB0706	TB0777	TB0842
TB0347	TB0422	TB0515	TB0581	TB0648	TB0709	TB0778	TB0843
TB0348	TB0423	TB0516	TB0582	TB0649	TB0710	TB0779	TB0844
TB0351	TB0424	TB0518	TB0583	TB0650	TB0712	TB0780	TB0846
TB0352	TB0427	TB0521	TB0584	TB0651	TB0714	TB0781	TB0847
TB0353	TB0429	TB0523	TB0585	TB0652	TB0718	TB0782	TB0848
TB0355	TB0437	TB0524	TB0588	TB0653	TB0719	TB0784	TB0849
TB0358	TB0438	TB0525	TB0590	TB0655	TB0720	TB0785	TB0850
TB0361	TB0441	TB0528	TB0591	TB0657	TB0722	TB0786	TB0852
TB0362	TB0442	TB0529	TB0592	TB0658	TB0723	TB0787	TB0853
TB0364	TB0448	TB0530	TB0593	TB0659	TB0724	TB0789	TB0855
TB0365	TB0449	TB0532	TB0594	TB0660	TB0725	TB0790	TB0856
TB0366	TB0451	TB0533	TB0595	TB0661	TB0726	TB0791	TB0858
TB0367	TB0453	TB0534	TB0596	TB0662	TB0727	TB0792	TB0860
TB0368	TB0455	TB0536	TB0598	TB0664	TB0728	TB0793	TB0862
TB0369	TB0456	TB0537	TB0599	TB0666	TB0729	TB0794	TB0863
TB0370	TB0457	TB0538	TB0600	TB0667	TB0731	TB0796	TB0864
TB0372	TB0459	TB0539	TB0601	TB0669	TB0732	TB0798	TB0865
TB0373	TB0460	TB0540	TB0603	TB0670	TB0733	TB0799	TB0866
TB0374	TB0462	TB0541	TB0605	TB0671	TB0734	TB0800	TB0871
TB0377	TB0463	TB0542	TB0606	TB0672	TB0735	TB0801	TB0872
TB0382	TB0466	TB0543	TB0607	TB0673	TB0736	TB0803	TB0873
TB0383	TB0467	TB0544	TB0608	TB0674	TB0739	TB0804	TB0875
TB0384	TB0469	TB0545	TB0610	TB0675	TB0740	TB0805	TB0876
TB0385	TB0471	TB0546	TB0611	TB0676	TB0742	TB0806	TB0877
TB0386	TB0472	TB0547	TB0612	TB0678	TB0743	TB0807	TB0878
TB0387	TB0473	TB0548	TB0613	TB0679	TB0744	TB0809	TB0880
TB0390	TB0474	TB0549	TB0615	TB0680	TB0745	TB0811	TB0881
TB0391	TB0475	TB0551	TB0616	TB0681	TB0746	TB0812	TB0882
TB0394	TB0477	TB0552	TB0617	TB0682	TB0748	TB0813	TB0887
TB0395	TB0478	TB0553	TB0618	TB0683	TB0749	TB0814	TB0891
TB0396	TB0479	TB0554	TB0619	TB0684	TB0750	TB0815	TB0892
TB0398	TB0480	TB0556	TB0620	TB0686	TB0751	TB0816	TB0894
TB0399	TB0481	TB0557	TB0621	TB0687	TB0752	TB0817	TB0896
TB0400	TB0482	TB0558	TB0622	TB0688	TB0753	TB0818	TB0898
TB0401	TB0486	TB0559	TB0623	TB0689	TB0754	TB0819	TB0899
TB0402	TB0489	TB0561	TB0624	TB0691	TB0756	TB0820	TB0900

TB0902	TB0966	TB1038	TB1106	TB1186	TB1249	TB1313	TB1364
TB0904	TB0968	TB1039	TB1110	TB1187	TB1250	TB1314	TB1365
TB0905	TB0969	TB1040	TB1111	TB1188	TB1251	TB1315	TB1366
TB0907	TB0972	TB1041	TB1112	TB1190	TB1252	TB1316	TB1367
TB0908	TB0973	TB1042	TB1113	TB1191	TB1253	TB1317	TB1368
TB0909	TB0974	TB1043	TB1115	TB1192	TB1254	TB1318	TB1369
TB0910	TB0975	TB1044	TB1116	TB1194	TB1256	TB1319	TB1370
TB0911	TB0977	TB1045	TB1118	TB1196	TB1257	TB1320	TB1371
TB0913	TB0978	TB1046	TB1119	TB1197	TB1258	TB1321	TB1372
TB0914	TB0979	TB1047	TB1122	TB1198	TB1259	TB1322	TB1373
TB0915	TB0980	TB1048	TB1124	TB1199	TB1260	TB1323	TB1374
TB0916	TB0981	TB1049	TB1125	TB1200	TB1261	TB1324	TB1375
TB0918	TB0984	TB1050	TB1126	TB1201	TB1262	TB1325	TB1376
TB0919	TB0985	TB1051	TB1127	TB1203	TB1263	TB1326	TB1377
TB0920	TB0988	TB1053	TB1129	TB1204	TB1264	TB1327	TB1378
TB0922	TB0989	TB1054	TB1130	TB1205	TB1265	TB1328	TB1379
TB0923	TB0990	TB1055	TB1131	TB1206	TB1266	TB1329	TB1380
TB0924	TB0991	TB1056	TB1132	TB1207	TB1267	TB1330	TB1381
TB0925	TB0992	TB1058	TB1133	TB1208	TB1268	TB1331	TB1382
TB0926	TB0993	TB1060	TB1134	TB1209	TB1270	TB1332	TB1383
TB0927	TB0994	TB1063	TB1135	TB1210	TB1273	TB1333	TB1384
TB0929	TB0995	TB1064	TB1137	TB1212	TB1274	TB1334	TB1385
TB0930	TB0996	TB1068	TB1138	TB1214	TB1276	TB1335	TB1386
TB0931	TB0997	TB1069	TB1139	TB1215	TB1277	TB1336	TB1387
TB0932	TB0998	TB1070	TB1140	TB1216	TB1278	TB1337	TB1388
TB0933	TB0999	TB1071	TB1141	TB1217	TB1279	TB1338	TB1389
TB0934	TB1000	TB1072	TB1143	TB1218	TB1280	TB1339	TB1390
TB0935	TB1001	TB1075	TB1146	TB1219	TB1281	TB1340	TB1391
TB0936	TB1003	TB1076	TB1147	TB1220	TB1282	TB1341	TB1392
TB0937	TB1004	TB1077	TB1148	TB1222	TB1283	TB1342	TB1393
TB0938	TB1005	TB1078	TB1149	TB1223	TB1284	TB1343	TB1394
TB0941	TB1007	TB1079	TB1153	TB1226	TB1285	TB1344	TB1395
TB0942	TB1008	TB1080	TB1155	TB1227	TB1289	TB1345	TB1396
TB0944	TB1009	TB1081	TB1156	TB1229	TB1291	TB1346	TB1397
TB0945	TB1010	TB1083	TB1157	TB1230	TB1293	TB1347	TB1398
TB0946	TB1011	TB1085	TB1158	TB1231	TB1294	TB1348	TB1399
TB0947	TB1013	TB1086	TB1159	TB1232	TB1295	TB1349	TB1400
TB0949	TB1015	TB1087	TB1161	TB1233	TB1296	TB1350	TB1401
TB0950	TB1020	TB1088	TB1162	TB1234	TB1298	TB1351	TB1402
TB0951	TB1021	TB1089	TB1163	TB1235	TB1299	TB1352	TB1403
TB0953	TB1022	TB1092	TB1164	TB1236	TB1300	TB1353	TB1404
TB0954	TB1024	TB1093	TB1165	TB1237	TB1301	TB1354	TB1405
TB0955	TB1027	TB1094	TB1166	TB1239	TB1302	TB1355	TB1406
TB0956	TB1028	TB1096	TB1169	TB1240	TB1303	TB1356	TB1407
TB0957	TB1031	TB1097	TB1173	TB1241	TB1305	TB1357	TB1408
TB0959	TB1032	TB1098	TB1174	TB1243	TB1306	TB1358	TB1409
TB0961	TB1033	TB1099	TB1175	TB1244	TB1307	TB1359	TB1410
TB0962	TB1034	TB1100	TB1180	TB1245	TB1309	TB1360	TB1411
TB0963	TB1035	TB1101	TB1181	TB1246	TB1310	TB1361	TB1412
TB0964	TB1036	TB1102	TB1183	TB1247	TB1311	TB1362	TB1413
TB0965	TB1037	TB1105	TB1185	TB1248	TB1312	TB1363	TB1414

TB1415	TB1466	TB1520	h2300.0	m1294.0	m1545.0	m2116.0	m2828.0
TB1416	TB1467	TB1522	h2375.0	m1295.0	m1559.0	m2128.0	m2898.0
TB1417	TB1468	TB1523	h2398.0	m13.0	m1560.0	m2129.0	m984.0
TB1418	TB1469	TB1524	h2438.0	m1300.0	m1570.0	m2130.0	m987.0
TB1419	TB1470	TB1525	h2463.0	m1304.0	m1615.0	m2131.0	m990.0
TB1420	TB1471	TB1526	h2581.0	m1307.0	m1638.0	m2132.0	m991.0
TB1421	TB1472	TB1527	h2754.0	m1309.0	m1700.0	m2134.0	m992.0
TB1422	TB1473	TB1528	h2760.0	m1310.0	m1702.0	m2136.0	m995.0
TB1423	TB1474	TB1530	h3004.0	m1315.0	m1703.0	m2137.0	mal010070.0
TB1424	TB1475	TB1532	h3005.0	m1317.0	m1704.0	m2138.0	mal010071.0
TB1425	TB1476	TB1533	h3033.0	m1319.0	m1726.0	m2139.0	mal010074.0
TB1426	TB1477	TB1534	h3094.0	m1324.0	m1728.0	m2140.0	mal010075.0
TB1427	TB1480	TB1535	h3360.0	m1325.0	m1734.0	m2141.0	mal010077.0
TB1428	TB1481	TB1536	h3361.0	m1340.0	m1759.0	m2142.0	mal010078.0
TB1429	TB1482	TB1537	h3367.0	m1373.0	m1762.0	m2144.0	mal010079.0
TB1430	TB1483	TB1538	h3986.0	m1374.0	m1780.0	m2145.0	mal010080.0
TB1431	TB1484	TB1539	m1004.0	m1381.0	m1782.0	m2191.0	mal010081.0
TB1432	TB1485	TB1540	m1007.0	m1384.0	m1787.0	m2192.0	mal010084.0
TB1433	TB1487	TB1541	m1008.0	m1389.0	m1816.0	m2193.0	mal010086.0
TB1434	TB1488	TB1542	m1009.0	m1392.0	m1822.0	m2198.0	mal010087.0
TB1435	TB1489	TB1543	m1010.0	m1393.0	m1848.0	m2199.0	mal010088.0
TB1436	TB1490	TB1544	m1011.0	m1397.0	m1893.0	m2203.0	mal010099.0
TB1437	TB1491	TB1545	m1012.0	m1400.0	m1895.0	m2204.0	mal010100.0
TB1438	TB1492	TB1546	m1017.0	m1402.0	m1906.0	m2206.0	mal010102.0
TB1439	TB1493	TB1547	m1021.0	m1404.0	m1913.0	m2211.0	mal010103.0
TB1440	TB1494	TB1548	m1022.0	m1410.0	m1914.0	m2218.0	mal010105.0
TB1441	TB1495	TB1549	m1023.0	m1415.0	m1926.0	m2248.0	mal010106.0
TB1442	TB1496	TB1550	m1024.0	m1416.0	m1928.0	m2249.0	mal010108.0
TB1443	TB1497	TB1551	m1025.0	m1417.0	m1929.0	m2259.0	mal010109.0
TB1444	TB1498	TB1552	m1026.0	m1418.0	m1932.0	m2276.0	mal010110.0
TB1445	TB1499	TB1553	m1029.0	m1422.0	m1948.0	m2277.0	mal010111.0
TB1446	TB1500	TB1554	m1030.0	m1424.0	m1955.0	m2278.0	mal010112.0
TB1447	TB1501	TB1555	m1034.0	m1425.0	m1956.0	m2323.0	mal010117.0
TB1448	TB1502	TB1825	m1210.0	m1429.0	m1959.0	m2324.0	mal010118.0
TB1449	TB1503	TB1827	m1212.0	m1431.0	m1961.0	m2342.0	mal010119.0
TB1450	TB1504	TB1829	m1213.0	m1438.0	m1967.0	m2343.0	mal010120.0
TB1451	TB1505	TB1831	m1215.0	m1439.0	m1973.0	m2346.0	mal010121.0
TB1452	TB1506	TB1834	m1221.0	m1441.0	m1976.0	m2350.0	mal010123.0
TB1453	TB1507	TB1835	m1232.0	m1444.0	m1978.0	m2352.0	mal010124.0
TB1454	TB1508	TB1836	m1233.0	m1448.0	m1979.0	m2360.0	mal010128.0
TB1455	TB1509	h1249.0	m1236.0	m1449.0	m2003.0	m2361.0	mal010129.0
TB1456	TB1510	h1329.0	m1253.0	m1452.0	m2006.0	m2376.0	mal010130.0
TB1457	TB1511	h1578.0	m1270.0	m1456.0	m2007.0	m2384.0	mal010131.0
TB1458	TB1512	h1580.0	m1271.0	m1458.0	m2010.0	m2402.0	mal010133.0
TB1459	TB1513	h1589.0	m1272.0	m1459.0	m2020.0	m2416.0	mal010134.0
TB1460	TB1514	h1856.0	m1273.0	m1461.0	m2021.0	m2435.0	mal010136.0
TB1461	TB1515	h1964.0	m1274.0	m1475.0	m2032.0	m2440.0	mal010137.0
TB1462	TB1516	h1996.0	m1283.0	m1481.0	m2084.0	m2442.0	mal020102.0
TB1463	TB1517	h2101.0	m1287.0	m1488.0	m2085.0	m2459.0	mal020107.0
TB1464	TB1518	h2102.0	m1288.0	m1520.0	m2086.0	m2479.0	mal020110.0
TB1465	TB1519	h2264.0	m1292.0	m1533.0	m2113.0	m2508.0	mal020120.0

mal020130.0
mal020131.0
mal020132.0
mal020135.0
mal020136.0
mal020138.0
mal020139.0
mal020141.0
mal020142.0
mal020144.0
mal020145.0
mal020146.0
mal020147.0
mal020148.0
mal020150.0
mal020151.0
mal020152.0
mal020156.0
mal020157.0
mal020160.0
mal020162.0
mal020165.0
mal020167.0
mal020172.0
mal020173.0
mal020174.0
mal020176.0
mal020179.0
mal020181.0
mal020182.0
mal020185.0
mal020186.0
mal020187.0
mal020192.0
mal020193.0
mal020194.0
mal020195.0
mal020196.0
mal020197.0
mal020199.0
mal020200.0
mal020201.0
mal020202.0
mal020205.0
mal020206.0
mal020208.0
mal020209.0
mal020211.0
