# Supplementary material for: Universal Lineage-Independent Markers of Multidrug Resistance in Mycobacterium tuberculosis
Source: Microorganisms. 2024 Jun 30;12(7):1340. doi: 10.3390/microorganisms12071340 (PMC11278869; doi:10.3390/microorganisms12071340)
Supplement: Supplementary file 1 [file microorganisms-12-01340-s001.zip › Suppl. Table S2.pdf]

Suppl. Table S2. Mtb polymorphic sites associated with multidrug resistance.

Location*	Locus tag or gene	In Codon	Found substitutions	Associations with antibiotic resistance†
1779274	Rv1572c	9	C > CGGGGTCGAGG, C > CGTGATCGAGGTG, C > CGTGGTCGAG, C > CGTGGTCGAGGTGG, C > CGTGGTGGAGGTGG, C > CGTGGTTGAG, C > CGTTGTCGAGGT, C > CTTGGTCGAG, C > G, C > T	AM, CM, EMB, INH, MFX, OFX, PTO, PZA, RIF, SM
1385255	PE_PGRS23	475	CG > C, C > CACCGGCGCC, C > CCCCGG, C > CCCCGGC, C > CG, C > CGCCCGCGCCG, C > CGCCG, C > CGCCGG, C > CGCCGGCG, C > CGCCGGCGCCG, C > CGCCGGCGCCGCCCG, C > CGCCGGG	AM, CM, EMB, INH, MFX, OFX, PTO, PZA, RIF, SM
1636170	PE_PGRS28	687	TTTCCGC > T, T > TCGC, T > TCGCCGCCCA	AM, CM, EMB, INH, MFX, OFX, PTO, PZA, RIF, SM
968434	PE_PGRS15	604	TGGCCGG > TGGCCGGGTGGGCCGG, T > TAACCGGGTT, T > TAGCCGGGCT, many others	AM, CM, EMB, INH, MFX, OFX, PTO, PZA, RIF, SM
1418868	Rv1269c	29	GCCAGGG > GCCAGGGACCCAGGG, A > AAGGAGCCA, A > ACGGAGCCA, A > ACGGCGCCA, A > ACGGGAGCCA, A > ACTGAGCCA, many others	AM, CM, EMB, INH, MFX, OFX, PTO, PZA, RIF, SM
859241	*cyp123*	278	CCTC > CCTCAGCTC	AM, CM, EMB, INH, MFX, OFX, PTO, PZA, RIF, SM
1191351	PE_PGRS20	267	G > C, G > GGACA, G > GGACAG, G > GGACGG	CM, EMB, INH, MFX, OFX, PTO, PZA, RIF, SM
968429	PE_PGRS15	606	C > CTGGTTGGCA, C > CGAGTTGCCCG, C > CGGATTGGCGGG, C > CGGCTTGCCCGG, C > CGGCTTGGGCGG, many others	AM, CM, INH, MFX, OFX, PTO, PZA, RIF, SM
3739978	PE_PGRS50	933	TG > TGAGGCCGGAG, T > A	AM, CM, EMB, MFX, OFX, PTO, PZA, SM
1189139	PE_PGRS19	429	CCGCCGGCCCCGCC > CCGCCGGCCCCGCCCTTGACGCCGGCCCCGCC, C > CCGCCGGCCCAGCCGTTGCCGCCGGCCC, C > CCGCCGGCCCCGCCCTTGACGCCGGCCCCGCC, C > CCGCCGGCCCCGCCGTTACCGCCGGCCCCGCCGTT, many others	AM, CM, EMB, INH, PTO, PZA, RIF, SM
3766912	PPE56	64	GCCGCC > GCCGCCCCCGCC, G > GCAGCCGC, G > GCCCCC, G > GCCCCCACC, G > GCCCCCCCC, G > GCCCCCGCC, G > GCCGCAGCCGC, G > GCCGCCACCGCC, many others	EMB, INH, MFX, OFX, PTO, PZA, RIF, SM
3899451	Rv3480c	318	TG > TGACG, T > TAGGACGACGG, T > TGACG, T > TGGACGACGG	AM, CM, EMB, MFX, OFX, PTO, PZA, RIF
1418870	Rv1269c	29	CAGGG > CAGGGAGCGAGGG	AM, CM, EMB, INH, MFX, PZA, RIF, SM
2061447	PE_PGRS33	410	CGGC > CGGCGCCGCTGGC, C > CACCGCCGCCG, C > CAGCACCGCC, C > CAGCCCCGCC, C > CAGCGCAGCC, C > CAGCGCCACC, C > CAGCGCCCCC, many others	AM, EMB, INH, OFX, PTO, PZA, RIF, SM
1636166	PE_PGRS28	689	G > -, G > GGCGCCACCCTTT, G > GGCGCGCCCC	AM, CM, INH, MFX, OFX, PTO, RIF, SM
2564374	Rv2293c	220	A > AA, A > AAA, AC > ACC, A > AT	AM, CM, EMB, INH, OFX, RIF, SM
2180650	Rv1928c	190	T > C, T > TGCC, T > TGCCG	CM, EMB, MFX, OFX, PTO, PZA, SM
334657	PE_PGRS3	552	G > A, G > GCGGACGCC, G > GTGGACGC	AM, CM, INH, MFX, OFX, PTO, PZA
335704	PE_PGRS3	203	CGCCGCCGGCGCCG > CGCCGCCGGCGCCGCCGGGGCCGCCGGCGCCG, C > CACCGACGGC, C > CACCGCCGGC, C > CCCCCCCGGC, C > CCCCGCCGCC, C > CCCCGCCGGC, C > CCCCGCCGGCGCCGCCGGCGCCGCCGGCGCCG, C > CCGATGAG, and others	AM, CM, INH, MFX, OFX, RIF, SM
673341	PE_PGRS7	859	C > CCGTTGCCGCCG, C > CCGTTGCCGGCG, CCGATGCCGT > C	AM, CM, EMB, MFX, OFX, PTO, PZA
49692	Rv0045c	83	CCT > C, C > A, C > C, C > CGTG, C > CTG, C > G	AM, CM, EMB, MFX, OFX, PTO, PZA
4326464	*ethA*	337	ATGT > ATGTGT	AM, CM, EMB, OFX, PTO, PZA, SM
2133472	Rv1883c	74	CAT > CATGCCGTCCCCTCGAATC > CAGGCCGTCACCGCGCA, C > CAGGCCGTCACCTCGGA, C > CATACCGTCACCTCCCATACCGTC, C > CATACCGTCACCTCGAATACCGTC, C > CATCCCGTCACCCCGCAT, C > CATCCCGTCACCTCGGAT, many others	CM, INH, MFX, OFX, PZA, RIF, SM
916674	Rv0823c	322	C > T	AM, CM, EMB, MFX, OFX, PTO, PZA
859243	*cyp123*	277	TCC > TCAGCTCGATCGACGCC, TC > TCAGCTCGATCGA, TC > TCGCTCG, TC > TCT	AM, CM, EMB, MFX, OFX, PTO, PZA
4408009	*gid*	65	A > ACCCC, A > C, A > G	AM, EMB, OFX, PTO, PZA, SM
335701	PE_PGRS3	204	C > A, C > ACAGCATGGC, CGGCGCCGC > CGGCGCCGCAGGCGCCGC, and others	INH, OFX, PTO, PZA, RIF, SM
335693	PE_PGRS3	207	G > A, G > ACTGCTGGCGACCCCGCCAGCGCCGCCGGCGCCGCCGTTGCCGATGAGC, G > C, G > CCGTTGCCGATGAGC, GGCGCCGCC > GGCGCCGCCAGCGCCGCC, and others	EMB, INH, MFX, OFX, PTO, SM
1189147	PE_PGRS19	427	C > CCCGCCGTTGCCGCCGGCA	CM, EMB, MFX, OFX, PTO, PZA
1986637	Rv1754c	12	T > TCTGAACC, T > TGTG, T > TGTGAACAGATG, T > TGTGAACC, T > TGTGAACCG, T > TGTGACCCG, T > TTTGAACC	AM, EMB, INH, PZA, RIF, SM
1385254	PE_PGRS23	475	CCG > CCGCCGGCG, C > CCACCGGCGCC, C > CCCCCGGC, C > CCCCCGGCG, C > CCG, C > CCGCCCGCGCCG, C > CCGCCG, C > CCGCCGG, C > CCGCCGGCG, C > CCGCCGGCGCCG, and others	AM, CM, MFX, OFX, PTO, PZA
3737112	PE_PGRS49	443	T > C, T > A, T > G	EMB, INH, PZA, RIF, SM
3738028	PE_PGRS49	138	G > T	EMB, INH, PTO, RIF, SM
3743401	Rv3346c	19	C > CAGCAG, C > CCAGCACGAACCCCACCGCGAAGATCGACCCAACCAG, and others	EMB, INH, PZA, RIF, SM
373368	PPE6	782	T > G, T > TGCTGACGCTGCCGGTG, T > TGTCGACGCTGCCGGCGT, T > TGTTGACACTGCCGGTGTT, T > TGTTGACCGCTGCCGGTGTT, T > TGTTGACGATGCCGGTGTTGACGCCGCCGGA, and others	AM, CM, INH, PZA, SM
3738024	PE_PGRS49	139	CGTTG > CG, C > CG, C > CGCCAATG, C > CGCCAATGTTG, C > CGCCG, C > CTCCAAT	EMB, INH, PZA, RIF, SM
3748717	PPE55	1490	T > C	AM, CM, MFX, OFX, PTO
1189430	PE_PGRS19	332	GTGC > G, G > CGGCGGT, G > G, G > GCGGTGCCGCCGCCGCCGCCGT, G > GTGCCGCCGCCGCCGCCGT, G > T, G > TCCACCGAATTGGCCGCCGTTGCCGGCACCGCCGGCGGT, G > TTGCCGGCACCGCCGGCGGT	EMB, INH, PZA, RIF, SM
2045207	PE_PGRS32	546	C > CGCCGCCGGCCCCGCCGAT, C > T	EMB, INH, PZA, RIF, SM
1075945	Rv0963c	52	G > A	AM, CM, INH, MFX, OFX
1715968	*mmpL12*	549	G > A	EMB, INH, PZA, RIF, SM
3462145	Rv3093c	207	A > AGGCGC	EMB, INH, PZA, RIF, SM
335686	PE_PGRS3	209	C > CCAGCGTT, C > CCCCGCCGCC, CGCCGCCGG > CGCCGCCGGGGCCGCCGG, and others	EMB, MFX, OFX, PTO, PZA
1189431	PE_PGRS19	332	T > C, T > CCACCGAATTGGCCGCCGTTGCCGGCACCGCCGGCGGT, T > G, T > GGCGGT, T > T, T > TGCC, T > TGCCGCCGCCGCCG, T > TGCCGCCGCCGCCGCCGCCG, T > TGCCGGCACCGCCGGCGGT, TGCC > T	EMB, ETH, INH, RIF, SM
4206010	*fadE36*	303	TTGAAATA > T	EMB, INH, PZA, RIF, SM
673318	PE_PGRS7	867	T > G, T > TCCCGCCGTG, T > TGCCGCCGTTGCCGCCG, T > TGCCGCCGTTGCCGCCGTTGCCGCCGTTGCCGCCG, T > TGCCGCCTTTGCCGCC, T > TGGG, T > TGGGG, T > TGGGGC	AM, CM, MFX, OFX, PZA
3766924	PPE56	60	G > A, G > GACGCCA, G > GACGCCG, G > GCC, G > GCCACCGC, G > GCCCC, G > GCCCCCCC, G > GCCCCCGC, G > GCCGACGC, G > GCCGC, GC > GCCGCCCC, and many others	EMB, INH, PZA, RIF, SM
3740557	PE_PGRS50	740	C > CATTCCC, C > CCC, C > CCCA, C > CCCC, C > CCCCCCC, C > CCCCCCCC, C > CCCCCCCCC, C > CCCCCCCCCCC, CGCC > CGCCCCGCC, and many others	AM, CM, MFX, OFX, PTO
2089090	PE_PGRS34	144	CCCGCCGTTGCCGCCGTTGCCGCCGTTGCCG > CCCGCCGTTGCCGCCGTTGCCGCCGTTGCCGCCGTTGCCG	CM, INH, PTO, RIF, SM
2133470	Rv1883c	75	CGCAT > CGCATGCCGTCACCTTGCAT, C > CCCATGCCGTCACCGC, C > CCCATGCCGTCCCCCC, C > CCCATGCCGTCCCCGC, C > CCCATGCCGTCGCCTG, C > CCCTC, C > CGCAGGCCGTCACCGCGCA, and many others	CM, EMB, INH, RIF, SM
3238123	Rv2923c	126	G > GACCGTG, G > GCCAG, G > GCCCG, G > GCCG, G > GTCCGT, GTGC > G	EMB, INH, PZA, RIF, SM
2133473	Rv1883c	74	AT > ATGCCGTCACCTCGCCT, A > ACGCCGTCACCTCGCG, A > AGGCCGTCACCGCGCA, A > AGGCCGTCACCTCGGA, A > ATACCGTCACCTCCCATACCGTC, and many others	EMB, INH, PZA, RIF, SM
2604165	*lppP*	20	ACA > ATCA, A > AAAGTGCCAA, A > AAAT, A > AAATCA, A > ACAATCA, A > ACAGTGACAACAGTG, A > ACAGTGCAAACAGTGC, and many others	EMB, INH, PZA, RIF, SM
3331366	Rv2975c	83	CG > C, , C > CC, C > CCA, C > CCC, C > CCG, C > CCGG	EMB, INH, PZA, RIF, SM
1189581	PE_PGRS19	282	CGCCGGCCCCGCCGGC > CGCCGGCCCCGCCGGCCCCGCCGGC	EMB, INH, PZA, RIF, SM
2180817	Rv1928c	134	A > ACC, A > ACCT, A > ACCTCGCCTTG, A > ACGCCTTG, A > AGCA, A > C, A > G, A > T, ATCGCCTTG > A	AM, CM, MFX, OFX, PTO
968423	PE_PGRS15	608	G > GTTAGCCGGA	AM, CM, MFX, OFX, PTO
1625329	*opcA*	13	C > T	AM, CM, MFX, OFX, PTO

1633538	PE_PGRS27	364	C > CACGGCCGCC, C > CANNNNNNNNNN, C > CATG, C > CATGGCC, C > CATGGCCGCC, C > CATGGCCGCCCC, and others	EMB, INH, PZA, RIF, SM
2534564	Rv2262c	330	AGC > A, A > AACCCTTCG, A > AAGCAGAAG, A > ACG, A > AGCGGC, A > AGG, A > AGTGCAGCGG, A > C, and others	AM, CM, MFX, OFX, PTO
2061433	PE_PGRS33	415	T > TCCGCCGGCG	EMB, INH, PZA, RIF, SM
968428	PE_PGRS15	606	C > CAGGGTTGGA, C > CAGGGTTGTC, C > CCGAGTTGCCCG, C > CCGGCTTGCCCGG, C > CCGGCTTGGGCGG, CCGGGTTGG > CCGGGTTGGACGGGTTGG, and many others	AM, MFX, OFX, PTO, SM
1632542	PE_PGRS27	696	C > CCTCGGCGCC, C > CG, C > CGCCG, C > CGT, C > CGTCG, C > CGTCGCCGCCG, C > CGTCGG, C > CGTCGGCGCCG, C > CGTCGGCGCCGTTGCCGCCGGCCCCACCG, CGG > CG, and many others	EMB, INH, PZA, RIF, SM
2729611	Rv2434c	317	T > TCAGATC, T > TCGGA, T > TCGGATCGG, T > TCGTATCG, T > G	AM, CM, MFX, OFX, PTO

*Locations of polymorphic sites are given as in the reference genome *Mycobacterium tuberculosis* H37Rv (NC_000962.3)

†Antibiotic abbreviations: amikacin (AM), capreomycin (CM), ethambutol (EMB), isoniazid (INH), moxifloxacin (MFX), ofloxacin (OFX), prothionamide (PTO), pyrazinamide (PZA), rifampicin (RIF), and streptomycin (SM)
